# The Ultrastructure of the Spectacle, Cornea and Conjunctiva of the Short‐Nosed Sea Snake *Aipysurus apraefrontalis* (Elapidae, Squamata) With Particular Reference to the Sub‐Spectacular Space and Its Contents

**DOI:** 10.1002/jmor.70177

**Published:** 2026-09-18

**Authors:** H. Barry Collin, Boyin Liu, Sarah Wilson, Jenna M. Crowe‐Riddell, Shaun P. Collin

**Affiliations:** ^1^ Department of Optometry and Vision Science University of New South Wales Kensington New South Wales Australia; ^2^ Bioimaging Platform La Trobe University Bundoora Victoria Australia; ^3^ School of Biological Sciences Adelaide University Adelaide South Australia Australia; ^4^ School of Agriculture, Biomedicine and Environment La Trobe University Bundoora Victoria Australia; ^5^ Max Planck Queensland Centre (MPQC) for the Materials Science of Extracellular Matrices Queensland University of Technology Brisbane Queensland Australia

**Keywords:** adnexa, conjunctival sac, glandular tissue, mucin, secretion, sub‐spectacular space, vesicles

## Abstract

The eyes of snakes possess an outer protective spectacle, continuous with the dermis, and an inner cornea, continuous with the scleral eyecup, that encloses a fluid‐filled space. While the spectacle and cornea provide transparency and contribute to the refractive power of the eye, little is known of their ultrastructure and role in maintaining the contents of the sub‐spectacular space. Using micro computed tomography, light microscopy and transmission electron microscopy, this study reveals that the conjunctival portions of the corneal epithelial and spectacular endothelial lining of the short‐nosed sea snake *Aipysurus apraefrontalis* possess high densities of microvilli to increase the surface area and aid in intra‐ and extracellular movement of nutrients and waste products, and at least two types of vesicles that contain what appear to be mucin fibrils and actin filaments, respectively, that may play a role in secretion of their contents into the sub‐spectacular space. The discovery of several ducts in the conjunctiva suggests that the Harderian gland may also deliver (non‐lipid‐bearing) fluid and granules to this space via several routes. The likely functions of the conjunctival secretory tissue are discussed in the context of maintaining the fluid within the sub‐spectacular space and the need to assist in the control of smooth eye movements beneath the immobile spectacle.

## Graphical abstract

Using micro computed tomography, light microscopy and transmission electron microscopy, this study reveals that the conjunctival portions of the corneal epithelial and spectacular endothelial lining in the eye of the short‐nosed sea snake *Aipysurus apraefrontalis* possess high densities of microvilli to increase the surface area and two types of vesicles that contain what appears to be mucin fibrils and actin filaments. The vesicles may play a role in secretion into the fluid‐filled sub‐spectacular space that likely assists in lubrication and the control of smooth eye movements beneath the immobile spectacle.

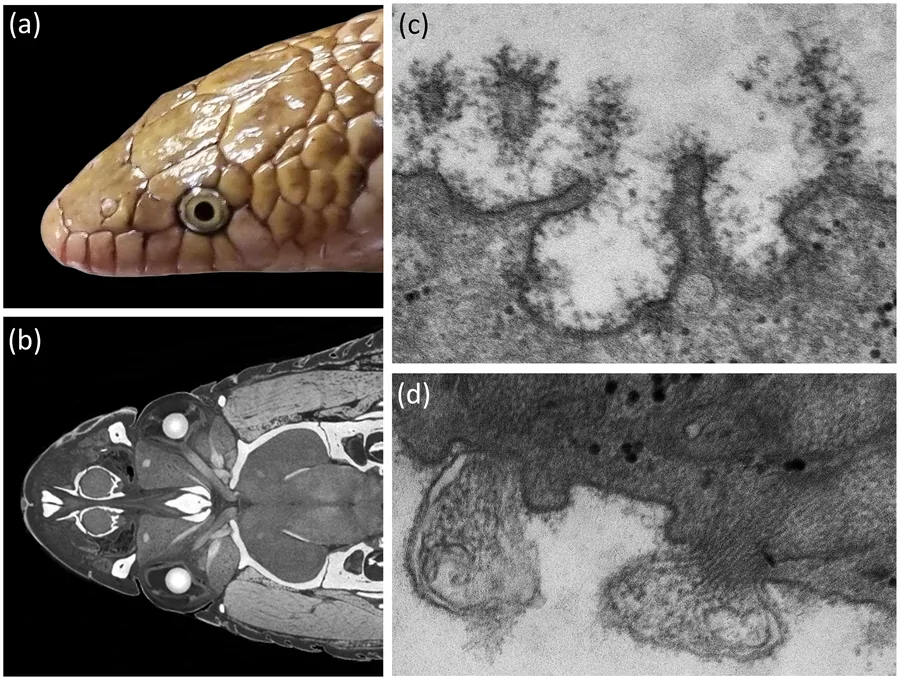

## 1 Introduction

The general structure of the anterior eye of snakes has been described by various authors (Kohl and Erster Thiel 1892; Neher 1935; Walls 1940, 1942), however, there are few studies of the ultrastructure of either the spectacle (Campbell et al. 1999; Da Silva et al. 2014, 2016; Collin et al. 2025) or the cornea. Formed by the embryonic fusion of the mesenchymal tissue that comprises the eyelids, the vascularised spectacle is continuous with the outer layers of the head scales (Schwartz‐Karsten 1933; Neher 1935; Bellairs and Boyd 1947) to form a protective, immobile ‘Brille’, which allows the underlying eye to rotate and the outer part of which is shed during periodic ecdysis. With a refractive index of approximately 1.5, the snake spectacle (and presumably the material filling the space between the spectacle and the cornea) constitutes the main refractive element of the eye (Van Doorn et al. 2014) and is thought to have evolved from burrowing lizards that needed to protect their eyes (Neher 1935; Bellairs 1948). This arrangement of the anterior eye, which is similar to the formation of a secondary spectacle in benthic and amphibious fishes (Walls 1942), appears to have a different origin from the primary spectacle in agnathan lampreys, where the cornea is divided into dermal and scleral corneas, derived from the surface ectoderm (Treviranus 1820; Franz 1934; Walls 1942; Duke‐Elder 1958; Collin et al. 2021). Although the surface topography has been examined using the scanning electron microscope (Campbell et al. 1999; Collin et al. 2025), the ultrastructure of the layers underlying the surface of the spectacle has received little attention (Da Silva et al. 2014, 2016). Similarly, the ultrastructure of the snake cornea has not been described, although Craig and Parry (1981) measured the corneal stromal collagen fibrils of the garter snake *Thamnophis sp*. and Pchelyakov (1982) reported on the embryology of the grass snake *Natrix natrix*, with few details and no micrographs of the morphology of the anterior segment. There also appear to be no studies of the ultrastructure of the conjunctiva in snakes.

Between the cornea and the spectacle in snakes is a sub‐spectacular space or conjunctival sac filled with fluid. The nature and source of this fluid have received considerable attention but have not been verified by ultrastructural studies. Among the first to examine the secretions associated with the snake eye was Kohl and Erster Thiel (1892), who studied two blind snakes, *Typhlops vermicularis* and *Typhlops braminus*, and reported that, at the edges of the conjunctival sac, the conjunctiva gradually becomes larger and wider, increasingly taking on the form of an aggregation of glandular cells. In the conjunctival fornix, a gland has actually developed from large multilayered cells, the secretion of which can be observed in the marginal parts of the conjunctival sac. Muhse (1903) reiterated the findings of Kohl and Erster Thiel (1892) for the same two snake species and also reported similar findings for *Typhlops lumbricalis*. Muhse (1903) also found that a Harder's gland is closely associated with the eye but that its function is doubtful and its secretions are not functional in connection with the eye. This last statement also echoes the previous findings of Kohl and Erster Thiel (1892), who had stated that, in these snakes, the Harderian gland has completely lost its connection to the eye and together with an altered tear duct, produces saliva or a similar secretion. In contrast, in the blind lizard, *Amphisbaenia punctata*, Payne (1906) reported that the secretion of the Harderian gland pours into the conjunctival sac.

Neher (1935) found that, at least in the Great Basin rattlesnake *Crotalus confluentus lutosus*, the ‘Brille’ or spectacle forms the external wall of the closed conjunctival sac and that this sac (later described as ‘the sub‐spectacular space’) is lined with an epithelium and is filled with fluid and claimed ‘its lubricatory gland (the Harder)’ has become a salivary gland. Walls (1942) also claimed that in snakes, the ‘Harderian gland secretion flows into the space between the delicate one‐layered corneal epithelium and the spectacle and drains through a duct in the nose’; however, he does not indicate the source of this information. Several other authors agree that among snakes, the lacrimal gland is lost and that its function is fulfilled by the Harderian gland, the secretion of which flows into the conjunctival sac or sub‐spectacular space (Bellairs and Boyd 1947; Chieffi et al. 1992; Payne 1994; Schwab 2012; Souza et al. 2015; Hellebuyck and Solanes Vilanova 2023). Walls (1942) also claimed that, in snakes, the Harderian gland has a single aperture, which opens into the conjunctival sac on the nasal side. Although Chieffi et al. (1992) found that a single secretory duct from the Harderian gland opens ventrally into the conjunctival fornix in *Coluber viridiflavus*, in other snake species, there may be several ducts (Bellairs and Boyd 1947; Payne 1994), some of which also provide lubricatory fluid to the vomeronasal organ (Rehorek 1997; Rehorek et al. 2000).

In their extensive study of many snake species, Bellairs and Boyd (1947) described the connections between the lacrimal gland, Harder's gland and the sub‐brillar (sub‐spectacular) space. The maturity of their specimens ranged from shortly before birth through to adults. They found several variations in these relationships. In ‘typical’ snakes, for example, *Natrix natrix* and *Thamnophis sirtalis* (Colubridae), *Bungarus fasciatus* (Elapidae*), Astrotia sp. (now Hydrophis)* (Hydrophidae*)* and *Vipera russelli* (Viperidae), known now as *Daboia russellii*, the Harderian gland discharges via a single duct directly into the upper end of the lacrimal duct, which is also connected with the anterior aspect of the sub‐brillar space by a single canaliculus and punctum. In the Boidae, i.e. *Constrictor constrictor* and *Trachyboa boulengeri*, the Harderian gland discharges into both a wide lacrimal canaliculus or duct and directly into the sub‐brillar space. There is also a small, but well‐defined, gland present, quite distinct from the Harderian, which is situated at the posterior pole of the orbit, that discharges directly into the postero‐medial aspect of the sub‐brillar space. This was not found in other species of snakes (Bellairs and Boyd 1947). In *Pareas carinatus*, a remarkable feature is a ring of secretory tissue, continuous with the anterior Harderian gland acini, which completely surrounds the upper end of the lacrimal duct and anterior part of the sub‐brillar space and discharges extensively into both (Bellairs and Boyd 1947).

Whereas ocular disease in squamates with a spectacle is infrequently seen in practice, disorders of the spectacle and the sub‐spectacular space are commonly encountered (Hellebuyck and Solanes Vilanova 2023). In order to apply an adequate diagnostic and therapeutic approach for these conditions, a sound knowledge and understanding of the anatomical and physiological peculiarities of the spectacle, sub‐spectacular space, and lacrimal drainage system are fundamental. The aims of this study are to describe the ultrastructure of the spectacle, the cornea and the conjunctiva of the short‐nosed sea snake, *Aipysurus apraefrontalis* (Elapidae), with special attention directed to the possible secretory role of the conjunctival epithelium lining the sub‐spectacular space.

## 2 Materials and Methods

### 2.1 Source of Animals

Three individuals of the short‐nosed sea snake, *Aipysurus apraefrontalis* (Elapidae, Squamata, Reptilia) were used in this study. This provided ocular tissue from a total of six eyes (spectacle, cornea, iris, conjunctiva, lens, limbus and angle including some of the surrounding scales) in addition to a micro computed tomographic scan (µCT) of the head and eyes. The remainder of the eyes (retina, optic nerve), other sense organs (olfactory and vomeronasal organs and the ears) and brains of these animals were all used for various other studies. Only adult snakes were collected from a trawl boat between 1 and 10 km off the coast of Exmouth in Western Australia, Australia in June 2023 with the approval of Adelaide University (formerly The University of Adelaide) Animal Ethics Committee (Science) (S‐2021‐017), the Western Australian Government (collection permit number FO25000393) and the Western Australian Department of Biodiversity, Conservation and Attractions (Section 40 Authorisation to take and disturb threatened fauna, TFA 2022‐0037). Trawled snakes are subject to injury including broken spines and general trauma. Sea snakes that are not injured but caught during trawling are promptly returned to the sea by workers on prawn fisheries in Exmouth. Our work with these Fisheries has led to a sea snake observer program to identify the species of any snakes caught by trawlers and the abundance of snakes within the trawled region. Their records of *A. apraefrontalis* are important since these indicate the only known breeding population for this species. All handling of snakes required personal protective equipment (PPE) given the venomous nature of these reptiles and all translocation, monitoring and euthanasia followed strict guidelines (Chapman et al. 2008). This study of injured individuals of short‐nosed sea snake, *Aipysurus apraefrontalis* [Smith, 1926] is opportunistic since this species is classified as critically endangered by the Australian Government (Department of Climate Change, Energy, the Environment and Water) and Western Australia (under the Biodiversity Conservation Act 2016). This species is endemic to Western Australia, and has been recorded from Exmouth Gulf, Western Australia (Storr et al. 2002) to the reefs of the Sahul Shelf, in the eastern Indian Ocean. The species was thought to prefer the reef flats or shallow waters along the outer reef edge in water depths from 1 to 10 m (Cogger 2000; Guinea 1993, 1995; McCosker 1975), although *A. apraefrontalis* has recently been identified to a depth of 67 m at Ashmore reef in Western Australia in 2021. Guinea and Whiting (2005) reported that this species may venture up to 50 m away from the reef flat. Recent work has shown that the Exmouth population represents a genetically and morphological distinct lineage from populations on the remote Timor reefs, which are associated with soft‐bottomed coastal habitats not coral reefs (Nankivell and Sanders 2026).

All snakes were euthanised with a lethal injection into the body cavity of diluted pentobarbitone (6 mg/mL) at a dosage of 150 mg/kg using a 23–30 G needle in accordance with the guidelines of the Australian Code of Practice for the Care and Use of Animals for Scientific Purposes, under Animal Ethics Committee protocols from Adelaide University (formerly The University of Adelaide) (S/2021‐017). The eyes and surrounding scales were fixed by immersion in either 4% paraformaldehyde in 0.1 M phosphate buffer (one eye of KLS1477) or Karnovsky's solution (2% paraformaldehyde and 2.5% glutaraldehyde in 0.1 M phosphate buffer) (other eye of KLS 1477 and both eyes of KLS 1490) for histology and light microscopy (using resin embedding) and transmission electron microscopy. Measurements of the sex, mass, snout to vent length, tail length and total length were recorded (where possible) using digital callipers or a tape measure prior to preservation. KLS1477 was a male 505 g with a 91 cm snout‐vent length (SVL), 9 cm tail length and 100 cm total length, while KLS1490 was a female with a 76.5 cm SVL, a tail length of 9.4 cm and a head length of 20 mm. The Western Australian Museum specimen (WAMR157818) used for µCT was an adult male, with a 77.7 cm snout‐vent length (SVL), 9.9 cm tail length and a head length of 17.2 mm. It was not possible to ascertain the stage of ecdysis for any of the three individuals, which we presume could have been different for each individual, or the influence of this process on the ultrastructure of the outer region of the spectacle. However, given the lack of replication of any of the outer layers of the spectacle, it is suspected that all three individuals were within an early phase of ecdysis and that the ultrastructural findings pertaining to the lining of the subspectacular space would not have been affected. We also acknowledge that given the small sample size, intraspecific variability and any systematic analysis of sexual dimorphism was not possible. However, all specimens were considered mature adults.

After immersion fixation for 24 h, the eyes, including the overlying ocular spectacle and surrounding scale tissue, were carefully dissected free of the orbits within the head. In two of the four eyes, the spectacle was carefully separated from the underlying cornea using a continuous radial incision in the conjunctival region of the eye at the level of the circumocular sulcus. For the remaining two eyes, the spectacle and cornea were left ‘attached’, thereby loosely maintaining the integrity of the sub‐spectacular space. All tissue was washed and stored in 0.1 M phosphate buffer (pH 7.4) with 0.05% sodium azide at 4°C until processing and analysis.

### 2.2 Micro Computed Tomography

A whole specimen of *A. apraefrontalis* from the Western Australian Museum that had previously been fixed in 10% formalin and stored in 70% ethanol was prepared for soft tissue micro computed tomography (µCT) scanning. The head was rehydrated over two to three days using a series of decreasing ethanol solutions (70%, 50%, 25%) before being immersed in 1.25% buffered Lugol's solution (I_2_ + KI + H_2_O + Na_2_HPO_4_ + KH_2_PO_4_) following Dawood et al. (2021) and Callahan et al. (2021). The head was µCT scanned as a region of interest scan using a Nikon XT H 225ST CT Scanner (Nikon Metrology, Tring, UK) at Flinders Microscopy and Microanalysis. The sample was scanned for 30 min with an X‐ray tube voltage of 150 kV and a current of 93 µA collecting 1300 2D x‐ray projections from different angles through a full 360° rotation of the sample with two times frame averaging applied. Tomographic projections were reconstructed using a filtered‐back projection algorithm and cross‐sectional images obtained using CT Pro 3D software (Nikon Metrology, Tring, UK). A median filter in 3D (radius 1) was applied in post‐processing.

### 2.3 Light Microscopy and Transmission Electron Microscopy

Two whole eyes (including spectacle, cornea, iris, conjunctiva and lens) and isolated pieces of the spectacle and cornea including transitions into the conjunctiva, skin (head scales) and scleral eyecup (of the remaining two eyes), were prepared for both light microscopy and transmission electron microscopy. For this analysis, tissue was post‐fixed in 1% (w/v) osmium tetroxide (Sigma‐Aldrich) in 0.13 mol l^−1^ Sorenson's buffer (0.13 mol l^−1^ Na_2_HPO_4_, Millipore Sigma, 0.13 mol l^−1^ KH_2_PO_4_, Sigma‐Aldrich, pH 7.4) for about 2 h. The tissue was rinsed in 0.13 mol l^−1^ Sorenson's buffer, dehydrated through a graded series of alcohols (25%, 50%, 70%, 80%, 95% and 100% EtOH) followed by immersion in 100% acetone, before infiltration with araldite (ProSciTech). Resin capsules were then cured at 60°C overnight. Transverse (semi‐thin) sections (1–2 μm) were cut from the resin‐infiltrated blocks using a Leica ultramicrotome EM UC7 (Leica Microsystems Pty Ltd, Macquarie Park, NSW, Australia) fitted with a 4‐mm histo‐diamond knife (ProSciTech). Sections were collected on glass slides and dried on a hotplate set to 120°C. Resin sections were mounted on subbed slides, stained with Toluidine blue, examined using a BH‐2 Olympus compound light microscope and photographed on an Olympus DP‐30 digital camera fitted with a trinocular C mount.

For transmission electron microscopy, ultrathin sections (70‐90 nm) were cut using a diamond knife (DiATOME 45°), collected on copper grids with 100 mesh or single slot (1 mm × 2 mm) (ProSciTech) and stained using lead citrate (Reynolds 1963) and uranyl acetate according to Collin et al. (2021). Sections were examined using a Jeol JEM‐2100 TEM with LaB_6_ filament operated at 80 kV and photographed on a AMT NanoSprint 15 Mk‐II CMOS camera.

### 2.4 Quantitative Analyses

The quantitative analyses of the morphological dimensions of the spectacle, cornea and conjunctiva and the size of the different secretory vesicles were primarily undertaken using digital images generated using transmission electron microscopy (TEM). Measurements were made manually on the computer images. The sample number for all features measured was high but varied (*n* = 28 to 96), thereby providing a robust mean and standard deviation (±SD). No assumptions of normality or homogeneity were assessed.

Quantitative data were compared using two‐tailed *t*‐tests for independent variables. The central and peripheral regions of the spectacle and cornea and the conjunctiva were analysed and both left and right eyes were compared. However, all features were found to be not significantly different for the two similar‐sized individuals and in left and right eyes, as has been found for ocular features in a range of other vertebrate eyes (Nam et al. 2006; Werther et al. 2017; Collin and Collin 2021). Therefore, the data for left and right eyes were pooled. No attempt was made to assess the degree of shrinkage in our study in order to allow direct comparison to be made with measurements derived using previously published transmission electron micrographs using similar methods. Shrinkage of around 30%–40% is typically expected following fixation and resin embedding for histology and TEM (Hayat 1986). Therefore, a correction factor should be applied to the data presented to give an estimate of the in vivo dimensions of the cellular components.

## 3 Results

**Figure 1:**
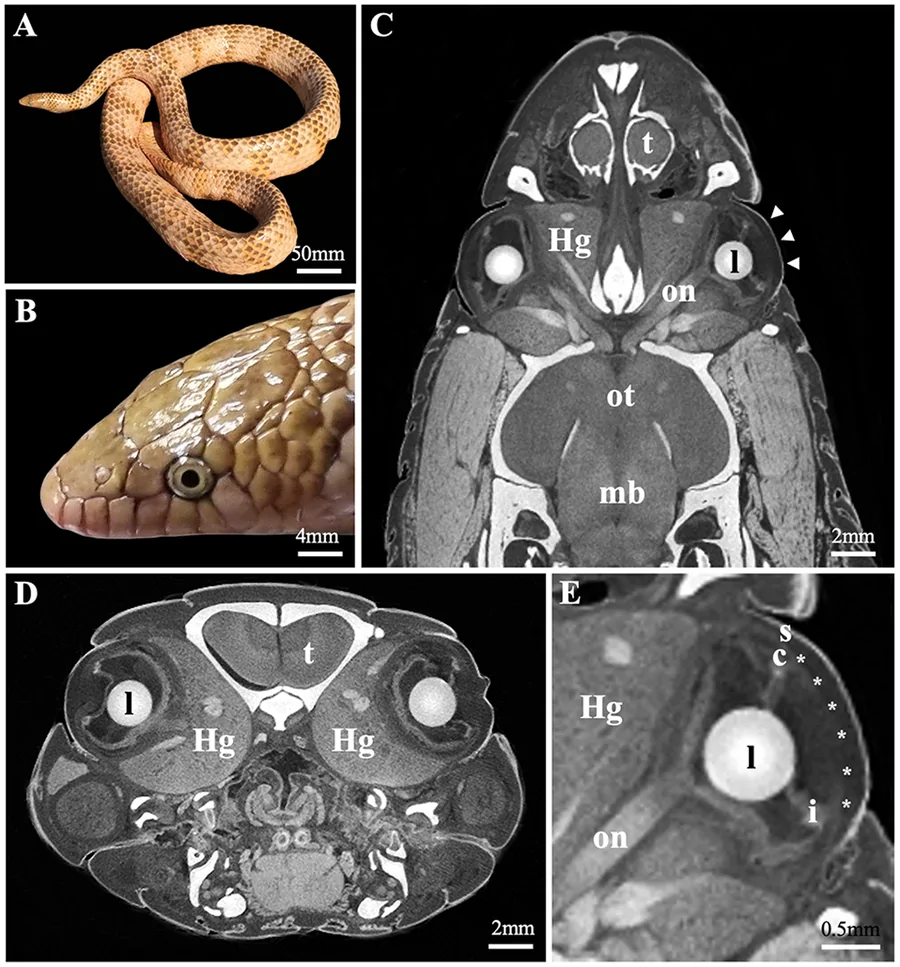
(A) Dorso‐lateral view of the short‐nosed sea snake *Aipysurus apraefrontalis* showing its mottled brown skin and small pointed snout. (B) Close up of the head of *A. apraefrontalis* showing the laterally‐placed hemispherical eyes. (C) Micro computed tomography generated (μCT) image in the horizontal plane showing the two laterally‐placed eyes. The convex spectacle (arrowheads) covering the eyes invaginates deeply around the circumference of the eyes, before it emerges to cover the head scales. Hg, Harderian gland; l, lens; mb, midbrain; on, optic nerve; ot, optic tectum; t, telencephalon. (D) μCT image of the head in transverse section showing the large Harderian glands (Hg) sitting medial of the two eyes. (E) Close up of the right eye shown in (C). The position of the thin sub‐spectacular space between the spectacle (s) and the cornea (c) is depicted by a series of asterisks. i, iris.

**Figure 2:**
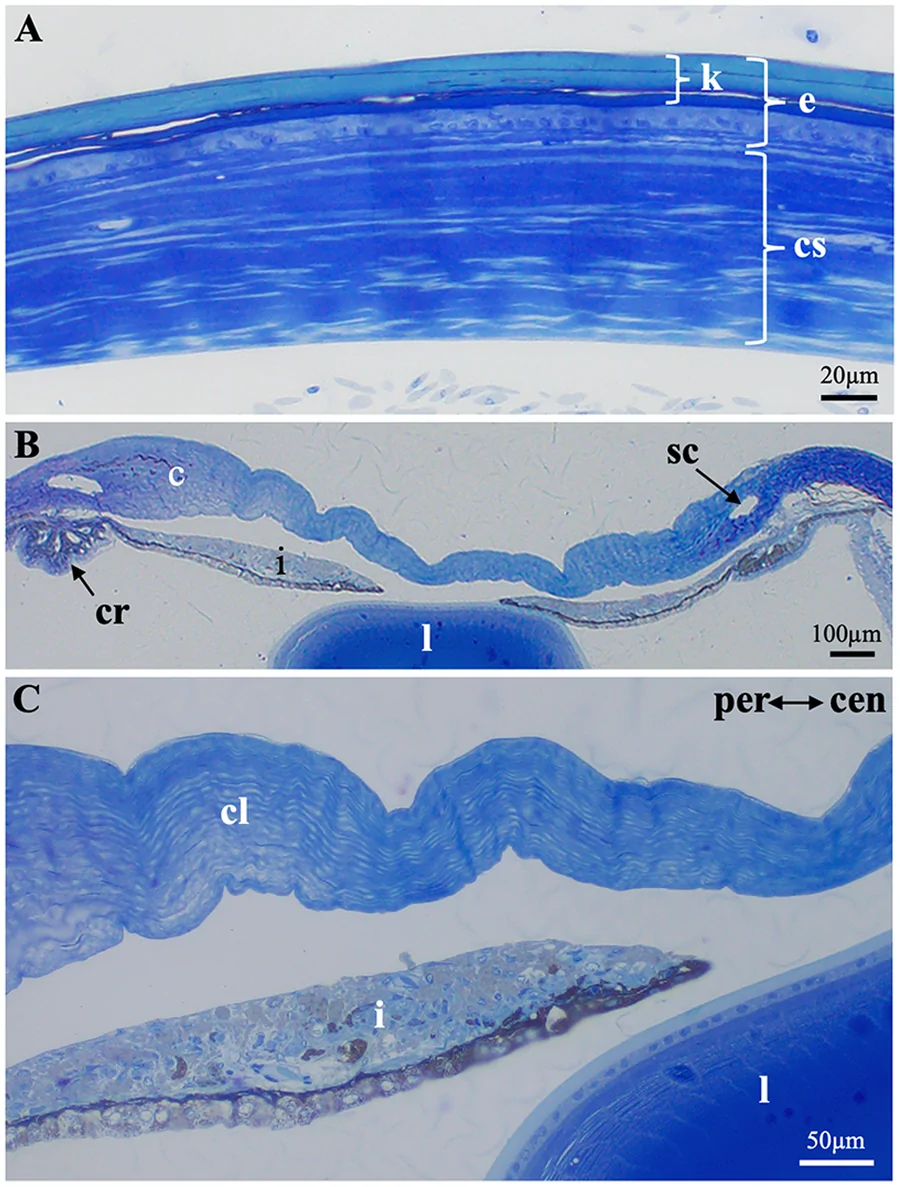
Anterior eye of *Aipysurus apraefrontalis*. (A) Transverse section through the central spectacle indicating the position of the outer keratinised layer (k) within a zone of epithelial cells (e) and a stroma containing collagen lamellae (cs). (B) Low power transverse section through the anterior segment of the eye beneath the spectacle. Note the thickening of the cornea (c) peripherally. (C) Higher power light micrograph of the cornea overlying the iris (i) and lens (l) showing the large number of collagen lamellae (cl). cen, centre; cr, ciliary roll; per, periphery; sc, canal of Schlemm.

Like most snakes, the eyes of the short‐nosed sea snake *Aipysurus apraefrontalis* are laterally‐placed with a light brown iris that matches its skin colour (Figure 1A,B). The eyes also possess a spectacle and a cornea separated by the sub‐spectacular space, which is lined with the spectacular endothelium anteriorly and the corneal epithelium, posteriorly (Figures 1C–E, 2A–C). Peripherally, the sub‐spectacular space is enclosed by conjunctival cell layers, which are continuous with the spectacular endothelium and the corneal epithelium.

### 3.1 The Spectacle

**Figure 3:**
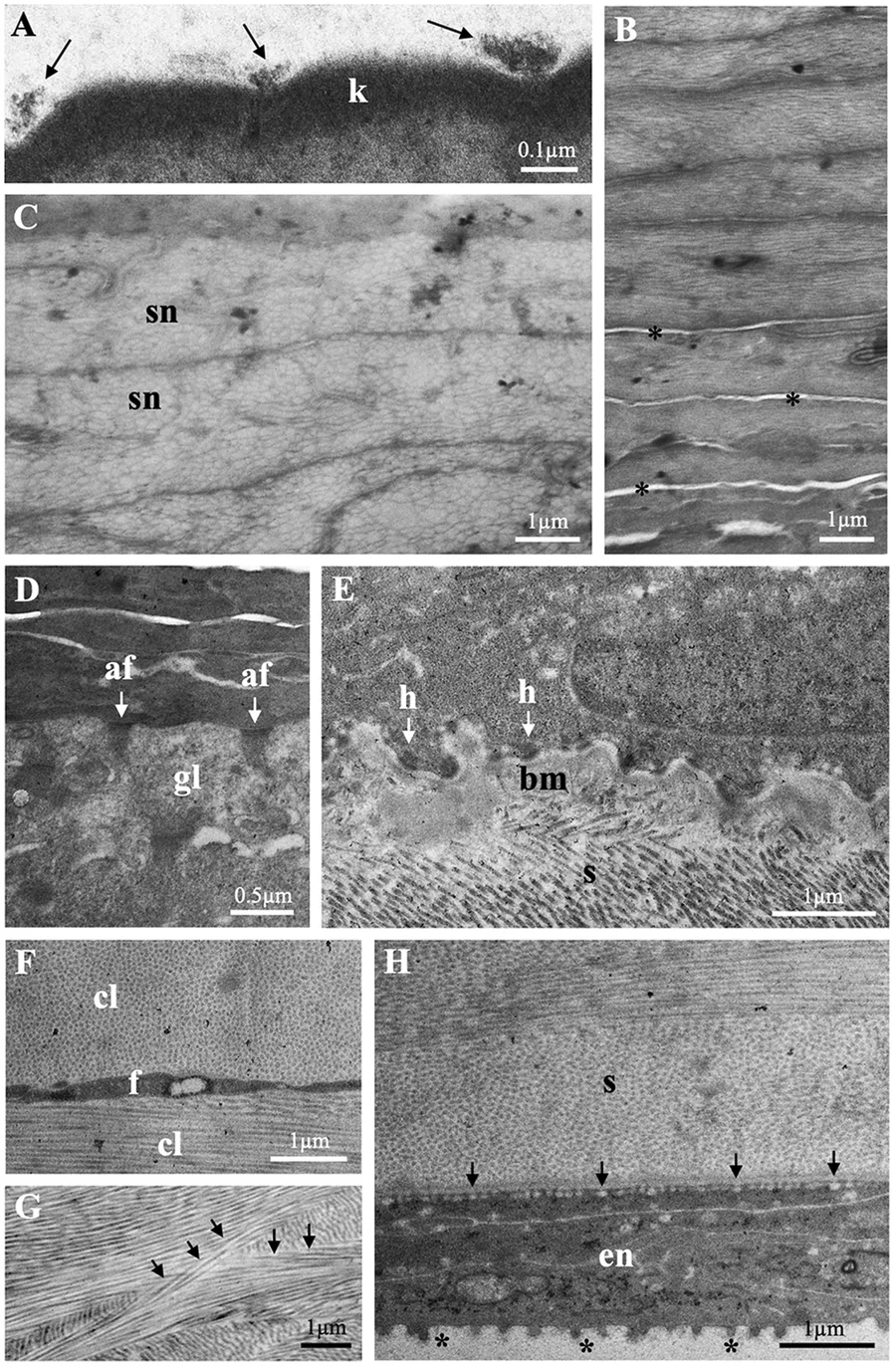
Ultrastructure of the spectacle. (A) Transmission electron micrograph of the outer keratinised layer (k) of the spectacle showing three indentations (arrows), all filled with debris. (B) Multiple layers of elongated keratinised cells, which lack organelles. Note the spaces between the cells (asterisks). (C) Close up of the fine spongey network (sn) within the cytoplasm of the elongated cells. (D) Immature alpha epithelial cells overlying a germinal layer (gl). Note the dense bundles of anchoring filaments (af) associated with their desmosome attachments on the internal (posterior) surface. (E) Area of transition from the epithelial cells to the stroma (s). Note the tortuous path of the basement membrane (bm) and the associated hemidesmosomes (h). (F) Fibroblast (f) in between two collagen lamellae (cl) each with a different (perpendicular) orientation. (G) High power micrograph of several bundles of collagen fibrils that branch and traverse to join similarly‐oriented lamellae (arrowheads). (H) Posterior border of the spectacle showing the transition from the stroma (s) to the single‐layered endothelium (en). Note the circular, electron‐lucent areas, some of which appear to be open to the space between the endothelium and the basement membrane (arrowheads). Large numbers of microvilli (asterisks) project from the surface of the endothelium into the sub‐spectacular space.

The thickness of the spectacle is approximately 48.6 µm in the centre and increases to around 114 µm in the periphery. The spectacle has three major components, namely an epithelium, stroma and an endothelium (Figures 2C and 3). The external layer of the epithelium, which in the skin is often referred to as the ‘oberhaütchen’, has small indentations in the surface, measuring 106 to 339 nm in diameter and up to 120 nm in depth. The majority of these indentations are filled with some form of debris (Figure 3A).

The superficial layers of the epithelium are made up of thin elongated keratinised cells, devoid of organelles, where the cell contents appear as a fine spongy network (Figure 3B,C). In the skin, this layer is often referred to as the beta‐layer (Lillywhite and Menon 2019). Next are several relatively electron‐dense layers of partially keratinised cells, equivalent to the mature alpha‐layers of the skin, followed by some layers of immature alpha‐cells, with numerous spaces present between the cells (Figure 3B,D). The basal epithelial cells form a germinal layer of large cells with large nuclei. The immature alpha‐cells are attached to the active germinal cells via desmosomes, which have dense bundles of anchoring filaments on the internal aspect but none are visible on the external or keratinising side (Figure 3D). In the cytoplasm of the peripheral epithelial cells adjacent to the basement membrane area are some scattered small (diameter of 65.4 ± 12.7 nm), circular, pale, electron‐lucent areas, some of which appear to be open to the space between the epithelium and the basement membrane (Figure 3E and 5). The basement membrane is irregular with a mean thickness of 97.3 ± 43 nm (range: 48 to 150 nm). It is thicker in the invaginations between the hemidesmosomes on the rather tortuous basal cell membranes (Figure 3E).

The main component of the spectacle is the stroma, which is composed of approximately 50 lamellae of parallel collagen fibrils with the fibrils of each lamella approximately at right angles to the adjacent lamellae (Figures 2A and 3F). The collagen fibrils have a diameter of 39.1 ± 5.9 nm (range: 25.4 to 50.8 nm) (Figure 3F,H). There is no Bowman's layer and the lamellae are thinner in the anterior stroma than in the central and posterior regions. There is occasional branching and anastomosing of the corneal lamellae (Figure 3). Between the lamellae are very thin, elongated cells, resembling fibroblasts, which appear similar to the keratocytes found in the cornea (Figure 3F).

Behind the stroma is a monolayer of endothelial cells, with a thickness of 1.29 ± 0.08 µm, and a thin basement membrane (thickness: 44.5 ± 9.3 nm) on the stromal side (Figure 3H). In the cytoplasm of the peripheral endothelial cells adjacent to the basement membrane are groups of small (80 to 95 nm), circular, electron‐lucent areas, some of which appear to be open to the space between the endothelium and the basement membrane (Figure 3H and 5A). In some areas, the density of these pale areas is around 9 per µm or 81 per µm^2^. We were unable to ascertain with certainty, whether these areas are membrane‐bound. On the posterior surface of this spectacular endothelium are very numerous microvilli, with a length of 109.9 ± 17.2 nm and a thickness of 106.3 ± 27.5 nm (Figure 3H).

### 3.2 The Cornea

**Figure 4:**
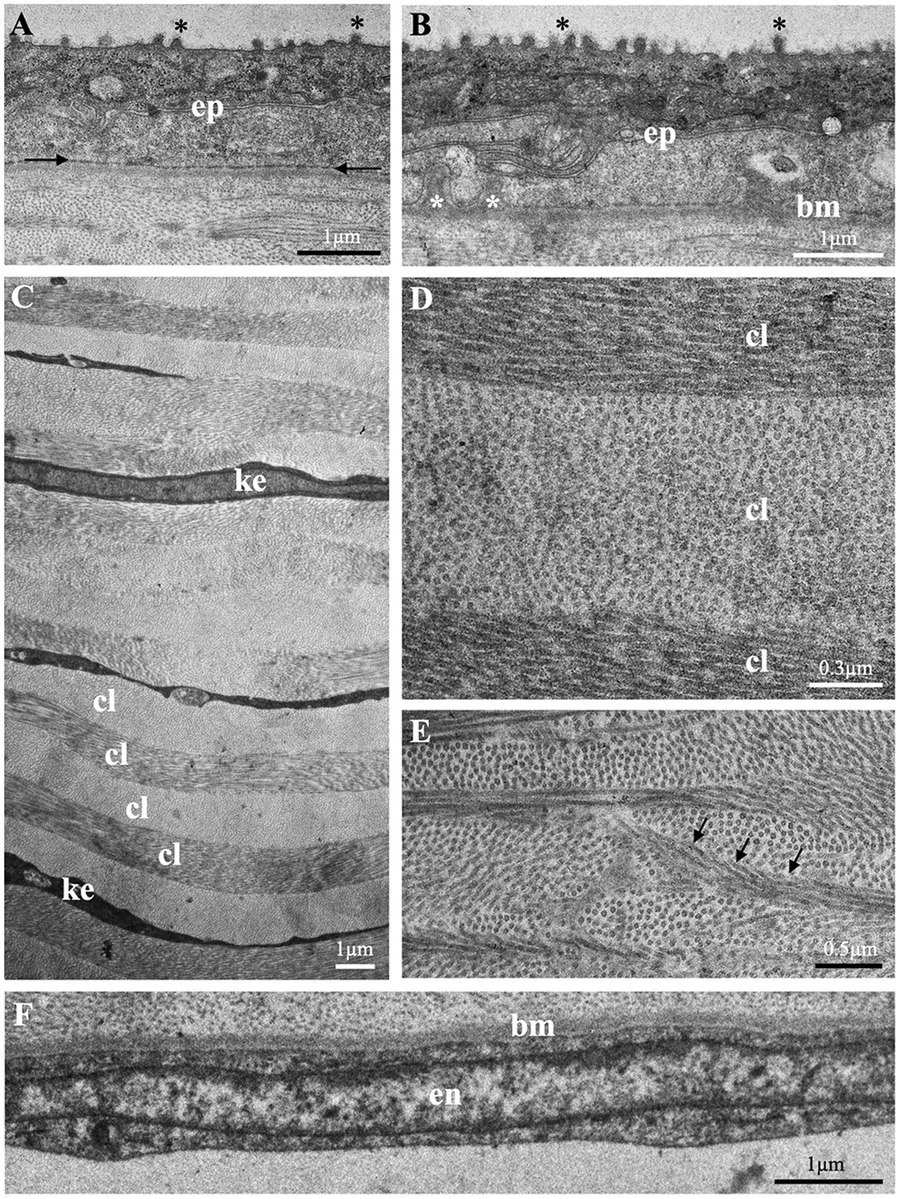
Ultrastructure of the cornea. (A and B) Transmission electron micrographs of the anterior, bilayered corneal epithelium (ep) showing numerous microvilli (black asterisks) projecting from the surface and the irregular basal cell membrane (arrows). Note that in some regions, invaginations of the basal cell membrane project anteriorly, where the basement membrane (bm) is much thicker (white asterisks). (C) Low power electron micrograph of the corneal stroma showing the layers of collagen lamellae (cl), each orientated perpendicular to its neighbour with a number of intervening keratocytes (ke). (D) High power of the stromal collagen fibrils, which comprise each collagen lamella (cl). (E) An example of small bundles of branching collagen fibrils (arrows). (F) Monocellular endothelium (en) underlying its associated basement membrane (bm). Note the absence of microvilli projecting into the anterior chamber.

**Figure 5:**
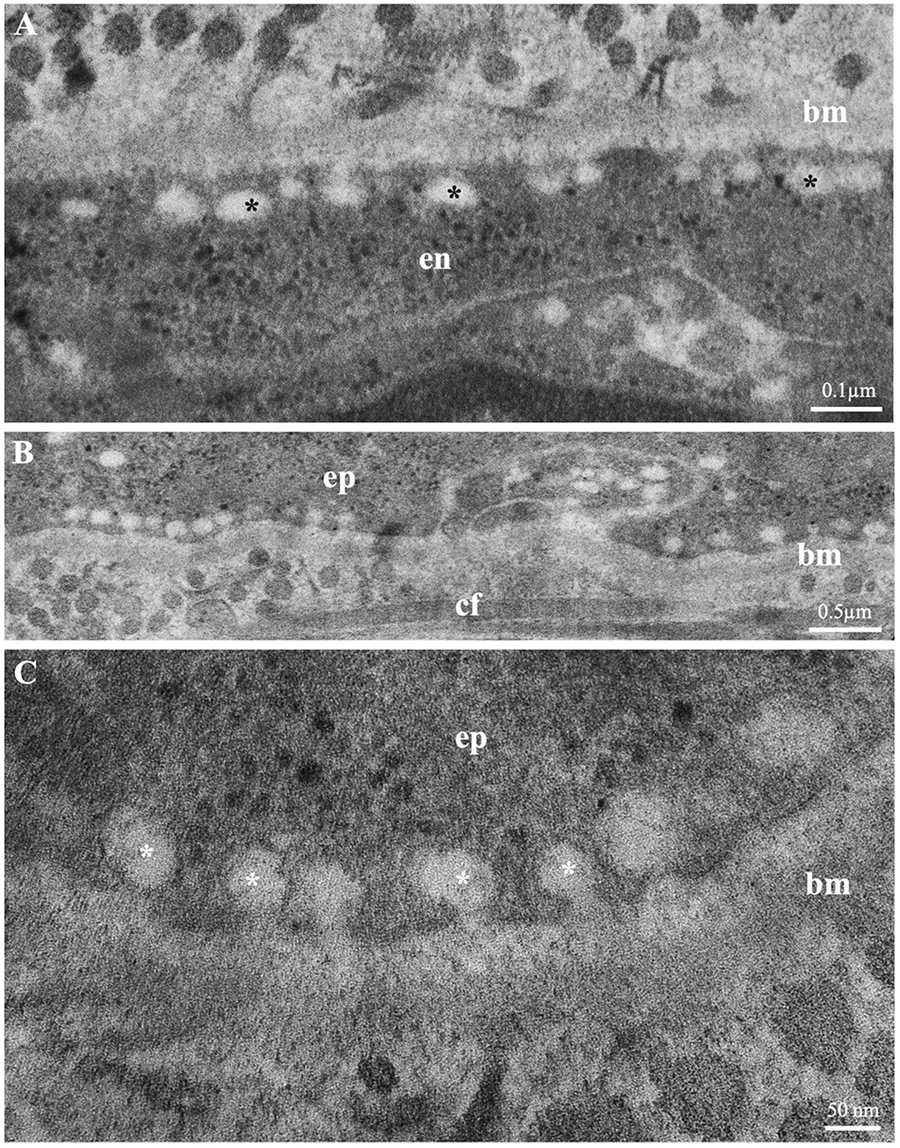
(A) Transmission electron micrograph of the basal cell membranes of the endothelial cells (en) of the peripheral spectacle showing several regularly spaced areas of reduced electron density (black asterisks). bm, basement membrane. (B and C) Examples of the circular profiles of reduced electron density lining the basal cell membrane of the epithelial cells (ep) in the peripheral cornea. cf, collagen fibril. (C) Close up of the circular profiles in the peripheral cornea. Note the presence of a cell membrane surrounding these profiles (white asterisks) could not be confirmed.

The cornea of *A. apraefrontalis* is similar in structure to many other vertebrates, in that it has an epithelium with its basement membrane, a stroma and an endothelium with its basement membrane. The cornea has a total thickness of 42.1 µm in the centre and around 135.7 µm in the periphery (Figure 2B,C). On the surface of the epithelium are numerous microvilli measuring 114.4 ± 36.4 nm in length and 118.1 ± 22.8 nm in width (Figure 4A,B). The epithelium consists of two layers of cells with a thickness of 2.1 ± 0.2 µm in the centre and 9.8 ± 11.9 µm in the periphery (Figure 2B,C). The basal cell membrane of the inner (posterior) layer of the epithelium has some invaginations of the cell membrane filled with basement membrane extending up to 0.5 µm into the cell (Figure 4B). As in the spectacle, in the peripheral areas, there are numerous, circular electron‐lucent areas in the cytoplasm of the epithelial cells, many of which appear to be associated with breaks in the cell membrane (Figure 5B,C). In some other areas, there are numerous apparent breaks in the cell membrane in the absence of the pale, electron‐lucent areas. Hemidesmosomes are numerous and there is a well‐developed basement membrane with a thickness of 90.8 ± 11.9 nm (Figure 4B).

The stroma consists of approximately 45 lamellae of parallel collagen fibrils with the collagen fibrils of each lamella being approximately at right angle to the neighbouring lamellae (Figure 4C). The diameter of the collagen fibrils is 27.5 ± 2.8, with a range of 21.1 to 33.8 nm (Figure 4D), which is significantly (*p* < 0.00001) smaller than the fibres of the spectacle. The collagen fibrils below the basement membrane form lamellae, indicating that there is no Bowman's layer. The lamellae are thinner anteriorly than in the posterior stroma and branching and anastomosing of the collagen lamellae are occasionally seen (Figure 4E). Between the collagen lamellae are numerous flattened keratocytes (Figure 4C).

Posterior to the stroma is a monocellular endothelial layer with a thickness of 0.77 ± 0.19 µm and without microvilli (Figure 4F). The basement membrane has a thickness of 96.8 ± 19.5 nm; however, it is not a Desçemet's membrane (Figure 4F).

### 3.3 Conjunctiva Lining the Anterior Spectacular Space

**Figure 6:**
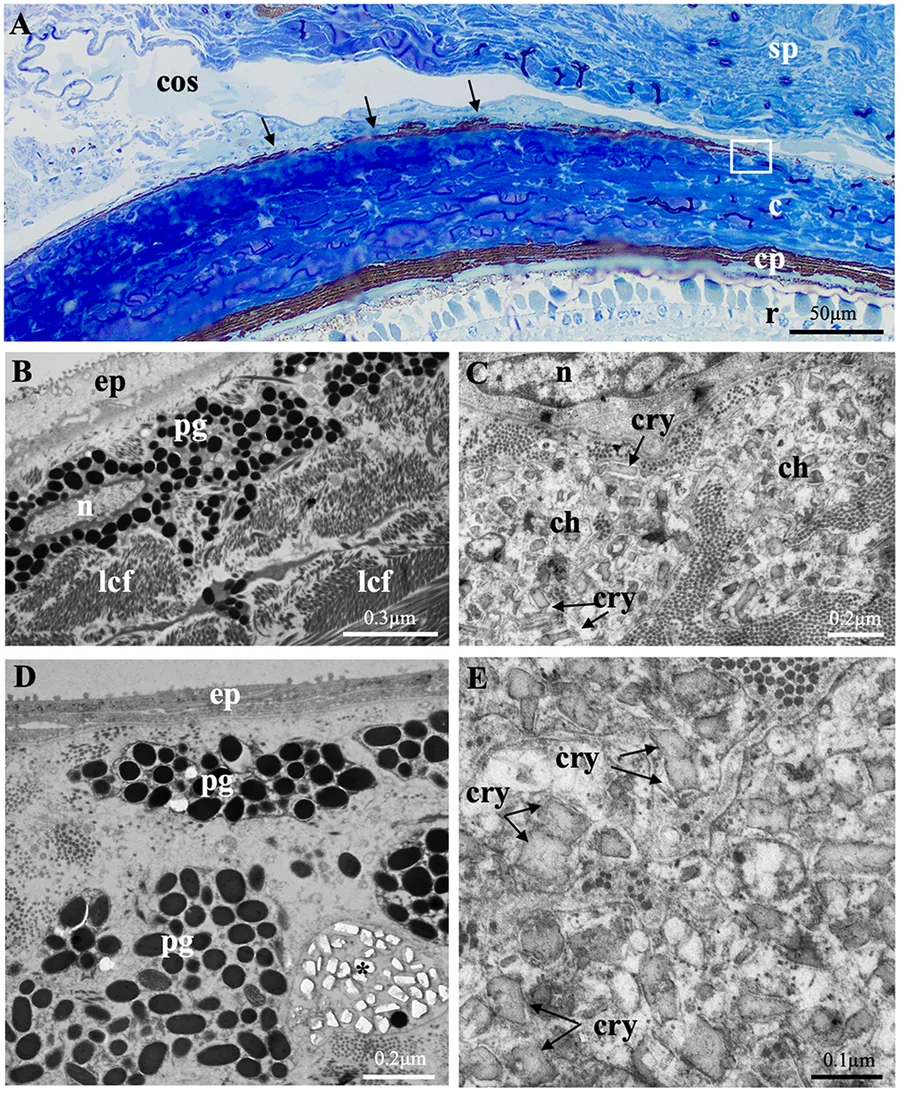
Ultrastructure of the conjunctiva. (A) Light micrograph of the conjunctival sac (cos), where the endothelial lining of the overlying spectacle (sp) meets the epithelial lining of the underlying cornea in the periphery of the eye (c). Note the presence of membrane‐bound cells containing osmiophilic (pigment) granules along the anterior border beyond the transitional zone of the cornea (arrows). cp, choroidal pigment; r, retina. (B and D) Transmission electron micrograph of the region boxed in (A) that shows dense aggregations of pigment granules (pg) underlying the epithelium (ep) interrupted by the profiles of large diameter collagen fibrils (lcf). In (D), note the contents of the crystalline structures (cry) in the nearby chromatophores (ch) have been lost during histological processing (asterisks). n, nucleus. (C and E). Low (C) and high (E) power electron micrographs of cells within the conjunctiva containing crystalline structures (cry) that may constitute iridophores.

On the anterior (spectacular) side, the conjunctiva lining the sub‐spectacular space consists of a single layer of endothelial cells, however, in the region of the fornix, where it joins the corneal epithelium, it increases in thickness, with two layers of cells (Figure 6A). There are numerous microvilli on the surface of the cells and on the stromal side is a basement membrane. In the basal cell membranes of the endothelial cells, there are several regularly spaced areas of reduced electron density, 80 to 95 nm in diameter (Figure 5A). Many of these areas appear to be open to the space between the cells and the basement membrane; however, the presence of a cell membrane surrounding these areas could not be confirmed. Whether these aid movement of substances in or out of the sub‐spectacular space in unknown.

Deep to the basement membrane is connective tissue and randomly‐oriented bundles of large collagen fibrils with a diameter of 87.5 ± 104.6 nm (range between 49.0 and 139.1 nm) (Figure 6B,C). These large fibres surrounding the spectacle are part of the transition to the skin, in which the collagen fibres may be much thicker, for example up to 127.0 ± 76.7 nm in the viper (*Vipera russelli*, recently renamed *Daboia russellii*) (Craig et al. 1987), and were used to confirm that the conjunctival observations were made beyond this transitional zone of the spectacle. Within the connective tissue of the conjunctival lining beyond the transitional zone of the cornea are numerous aggregations of membrane‐bound pigment cells containing osmiophilic granules, loosely orientated perpendicular to the incident light that would strike the outer regions of the eye and surrounding scales (Figure 6B,D). Often in conjunction with the deposits of pigment, are large chromatophores containing some crystalline components, which may reflect light (thereby constituting iridophores) given their shape and guanine‐like patterns (Figure 6C,E). Blood vessels and nerves are occasionally seen.

**Figure 7:**
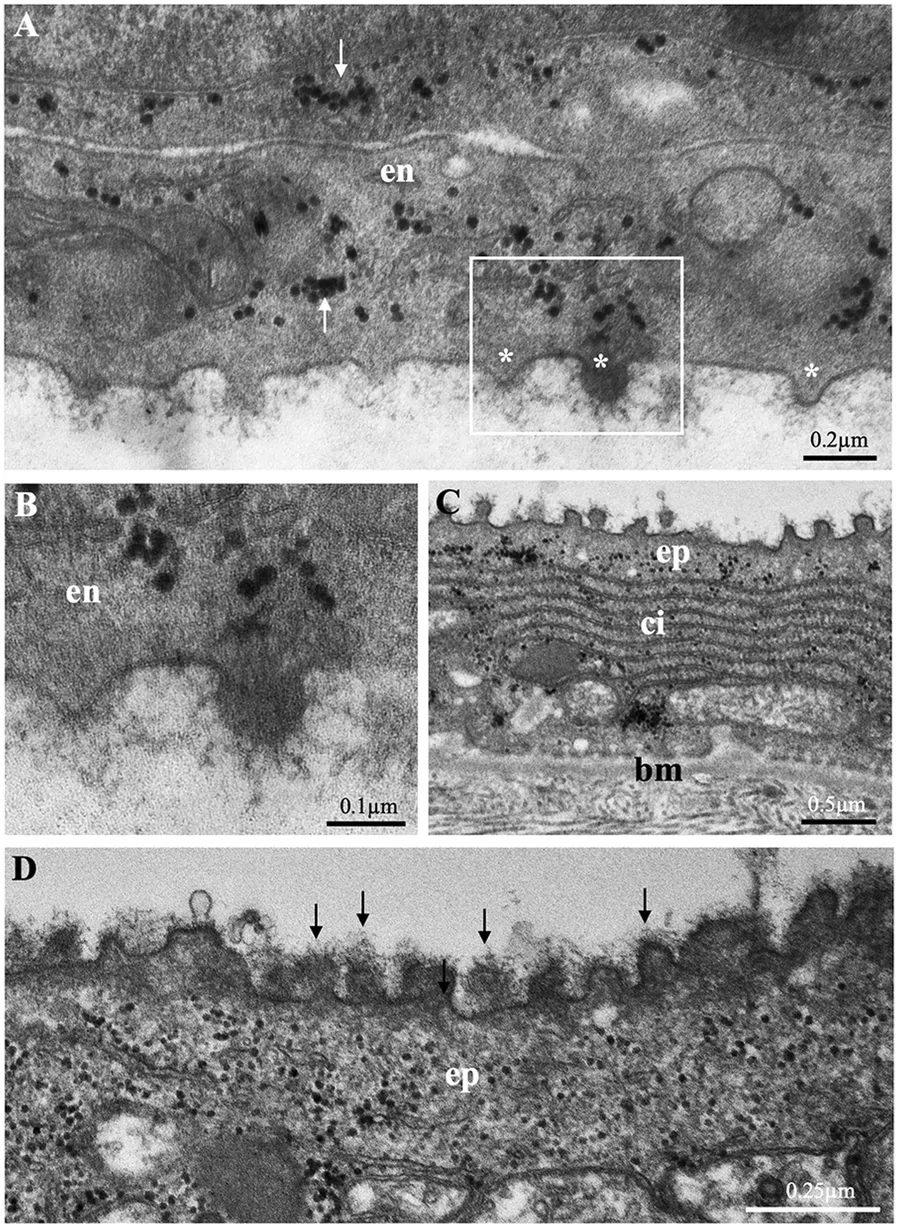
The lining of the sub‐spectacular space. (A and B) Electron micrographs of the inner surface of the endothelium (en) of the conjunctiva lining the anterior spectacular space showing a number of microvilli (asterisks) that are covered in short fibres. Note the osmiophilic glycogen granules within the cytoplasm (white arrow). (B) Close up of microvilli (outlined by the box in (A) with attached short fibres. (C) Epithelium (ep) of the conjunctiva lining the posterior spectacular space showing the complex of interdigitations (ci) between opposing cells. bm, basement membrane. (D) High power of the glycogen rich posterior conjunctival epithelial cells (ep) showing large numbers of microvilli covered in short fibres (arrows).

**Figure 8:**
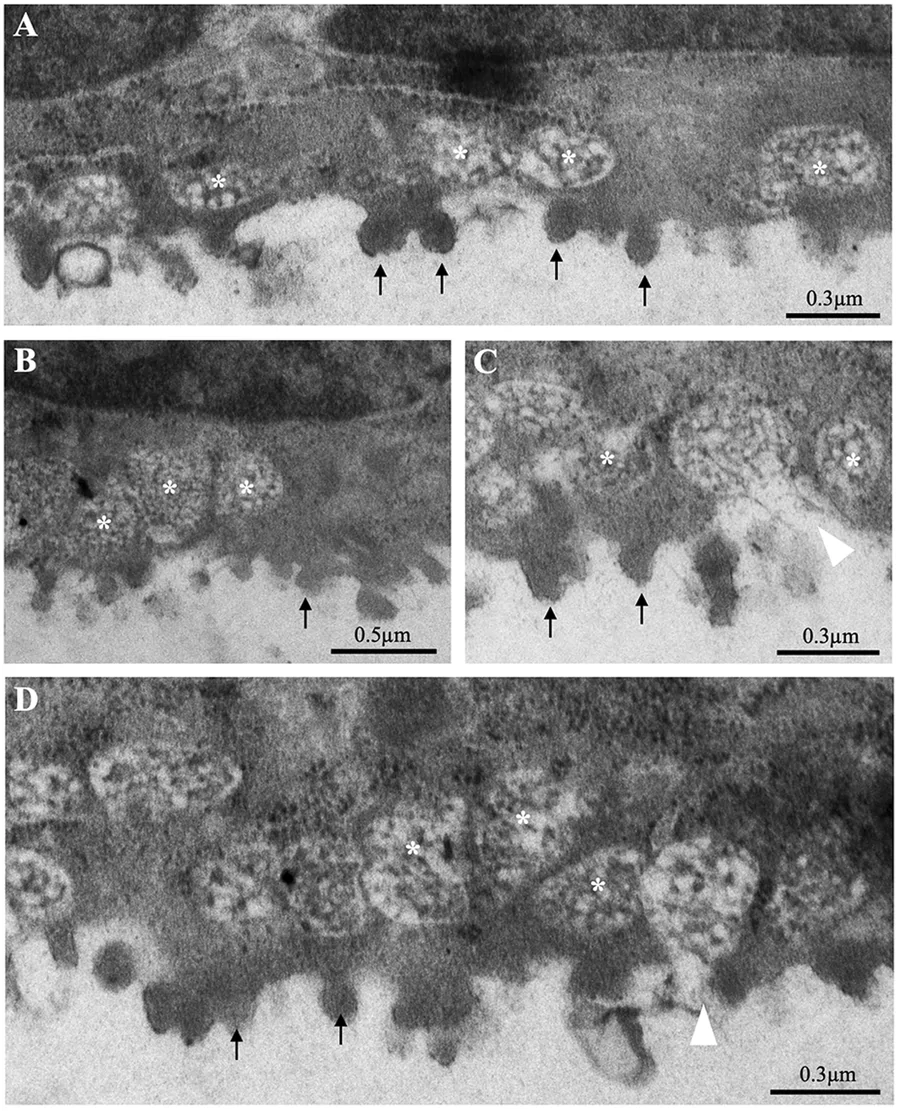
(A–D). Examples of Type I vesicles within the conjunctival endothelium lining the anterior spectacular space. Note the mottled appearance and electron dense contents of the vesicles (asterisks), which lie beneath the endothelial cell surface membrane and just anterior of numerous microvilli (arrows) (A and B). Several of these vesicles are open to the surface and are expelling material into the subconjunctival space (white arrowheads) (C and D).

**Figure 9:**
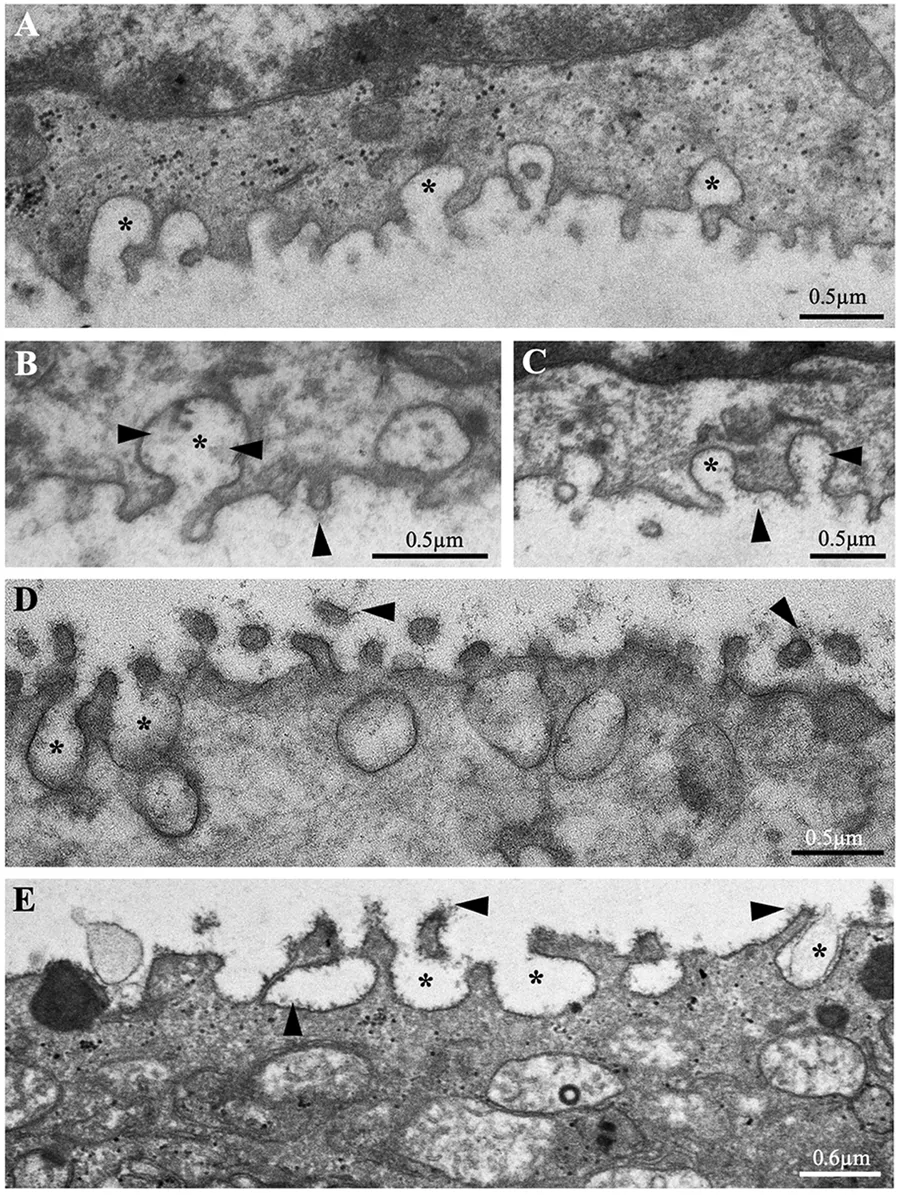
(A–C) Numerous Type I vesicles within the conjunctival endothelium lining the anterior spectacular space following the expulsion of their contents (asterisks). Note the small particles remaining around the perimeter of the open vesicles and attached to the microvilli (arrowheads). (D and E) Type I vesicles within the posterior conjunctival epithelium lining the subconjunctival space. Note that some vesicles are filled with small particles, while others (asterisks) are open to the surface.

**Figure 10:**
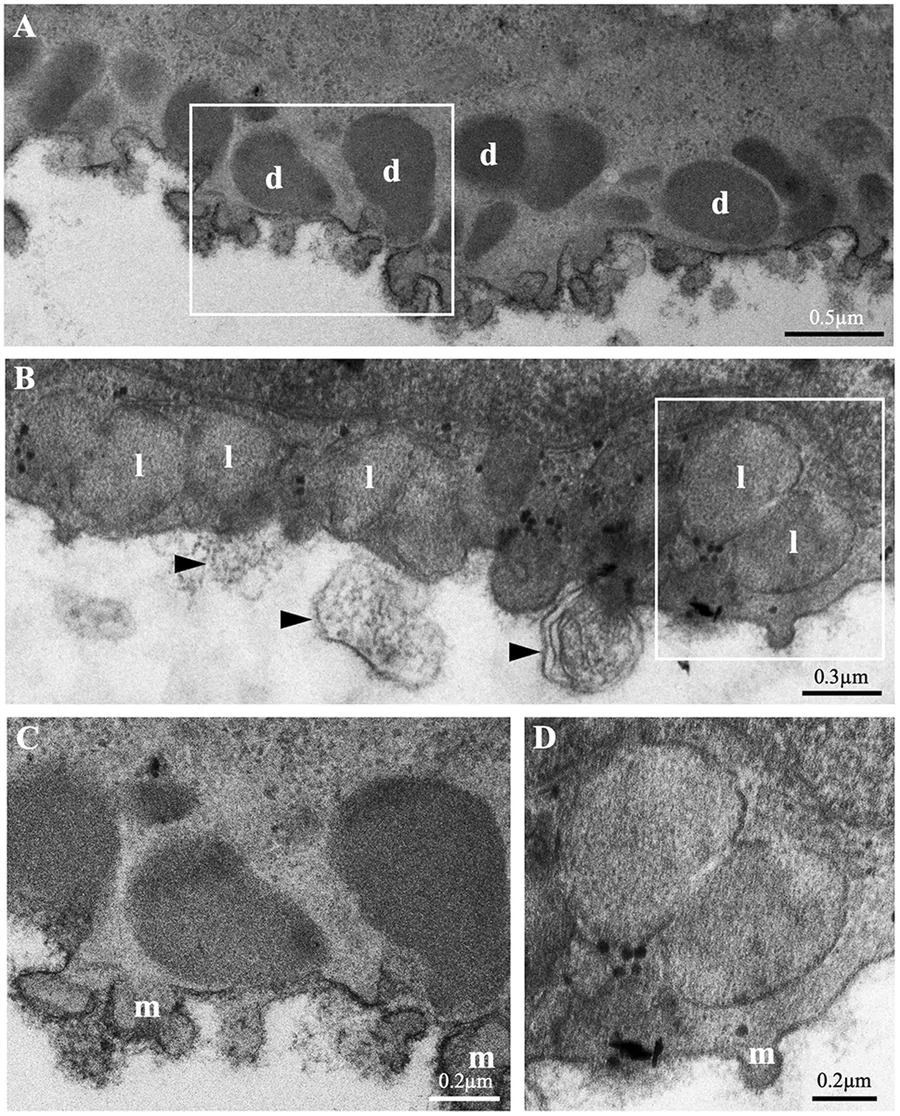
Type II vesicles within the conjunctival endothelium lining the anterior spectacular space. (A) Dark Type II vesicles (d), which appear as electron dense globules near the surface of the endothelium. (B) Lighter Type II vesicles (l), which are less electron dense, some of which are in the process of expelling their contents (black arrows). (C) Close up of the dark Type II vesicles boxed in (A) showing their granular contents and proximity to the surface membrane. (D) Close up of the light Type II vesicles boxed in (B). m, microvilli.

**Figure 11:**
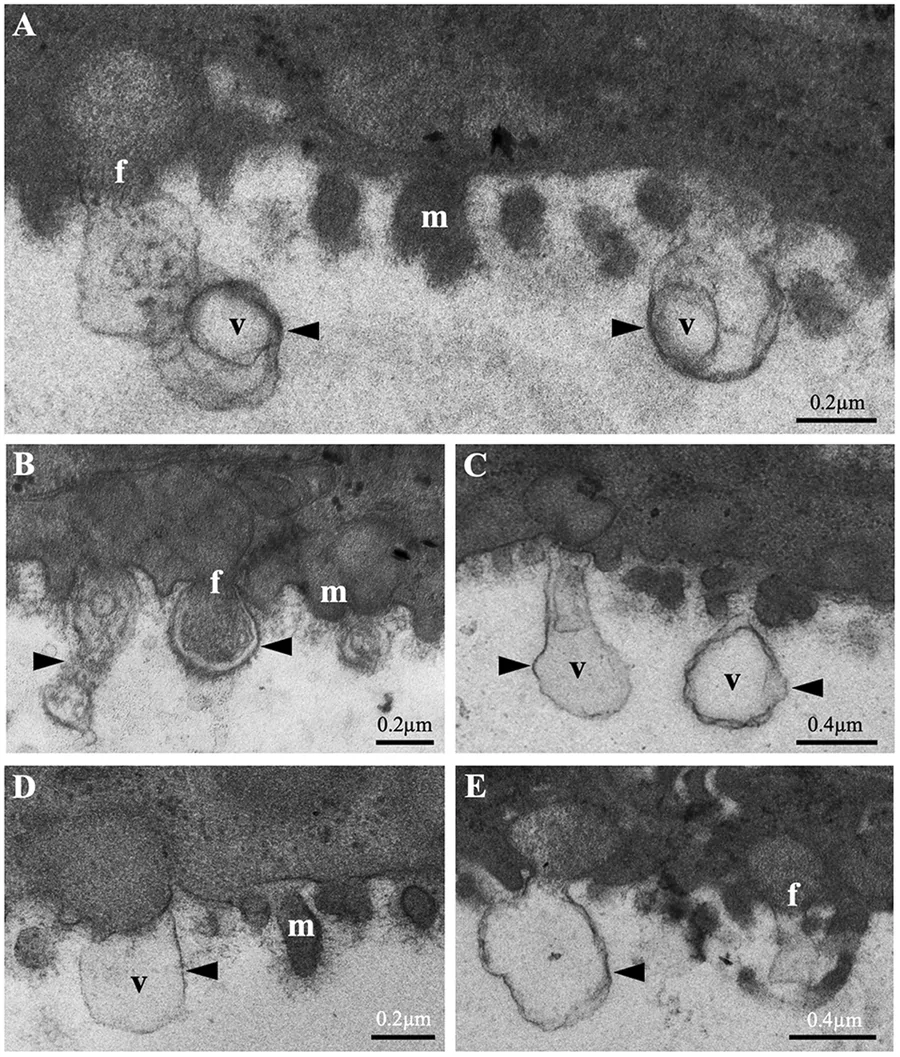
(A–E). Type II vesicles within the conjunctival endothelium lining the anterior spectacular space in the process of expelling their contents (black arrowheads). Note that some vesicles contain either long filaments (f) or appear as vacuoles (v), which may be attached to the membrane or free‐floating. m, microvilli.

**Figure 12:**
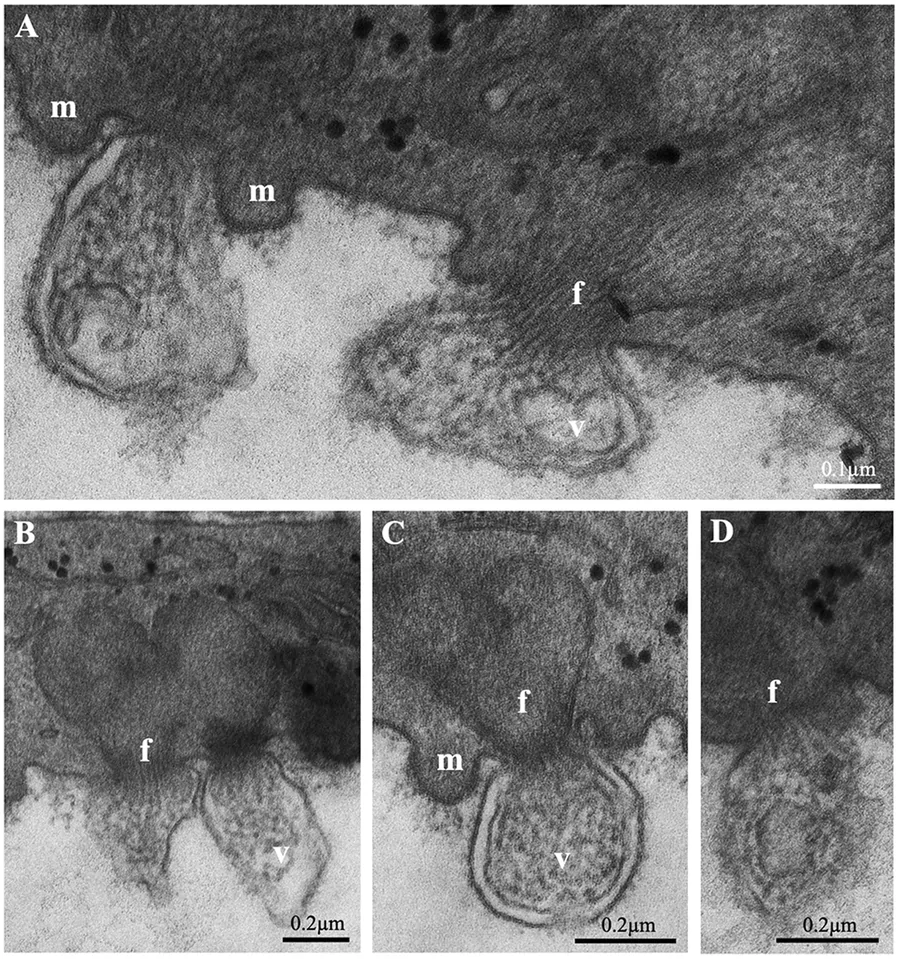
(A–D) High power transmission electron micrographs of Type II vesicles, which possess membrane‐bound vacuoles (v) attached to the cell surface and are filled with long filaments or fibres (f) that extend deep into the cell. m, microvilli.

At higher magnification, both the spectacular endothelium and corneal epithelium lining the sub‐spectacular space are rich in glycogen granules, have tortuous intercellular interdigitations and the surface is covered with numerous microvilli (Figure 7A–D). Within the conjunctival lining, the microvilli are covered in short fibres. Scattered throughout the endothelium of the conjunctiva lining the anterior spectacular space are cells with several types of small membrane‐bound vesicles, indicating a possible secretory function. These cells appear to be scattered throughout the endothelium, in that, they always appear to be surrounded by cells without vesicles. Based on their ultrastructure, at least two types of vesicles are characterised:

1. Vesicles with a mottled (non‐homogenous) content, associated with variations in electron density and a diameter of 335.1 ± 75.5 nm, are aggregated below the endothelial cell surface membrane (Figures 8A–D and 9A–C). On or near the surface of some of the cells are vesicles, which are mostly electron lucent, that is, they appear almost empty, with occasional electron‐dense components (Figure 9A–C). Many of these vesicles are open to the surface of the endothelium and appear to be expelling material into the sub‐spectacular space (Figures 8C,D and 9A–C). At this stage, there are numerous small particles, approximately 5 nm in diameter and up to 140 nm in length, which are attached to the inner part of the vesicle, to the outer surface of the endothelial cells and to the neighbouring microvilli (Figure 9B,C). These vesicles have a diameter of 331.2 ± 80.3 nm, which is not significantly different (*p* = 0.813) from the mottled vesicles and are thought to be the same structures but in a different phase. These vesicles (in both phases) are characterised as Type I.
2. A second type of vesicle (Type II) is present in the endothelial cells of the peripheral spectacle, although there may be two ultrastructural manifestations of these vesicles (Figure 10). These manifestations are primarily based upon electron density and release of contents. In one of these types of vesicles, the contents are electron dense and the vesicles have a diameter of 380.1 ± 111.4 nm and hence, are slightly larger than both of the Type I vesicles (*p* < 0.039). Some of these vesicles are attached to the cell membrane and appear to be discharging their contents into the sub‐spectacular space (Figure 10A,C, 11, and 12). The other group is lighter in colour (less electron dense), with a diameter of 389.1 ± 98.0 nm, which is not significantly different (*p* = 0.683) from the darker version but is larger (*p* < 0.0035) than both of the Type I vesicles (Figure 10B,D). Some of these lighter vesicles are in the process of expelling their contents into the sub‐spectacular space and this can be seen in two stages. Some membrane‐bound vesicles/vacuoles attached to the cell surface appear to contain long filaments measuring 5.7 ± 2.3 nm in diameter and up to 500 nm or more in length (Figures 11 and 12). These fibres extend deep into the cell. Next, there are large (up to 560 nm in diameter), membrane‐bound vesicles/vacuoles either attached to the cells or floating freely in the sub‐spectacular space. These structures are electron lucent and do not appear to contain any of the long filaments previously seen within the cell vesicle during the expulsion process (Figure 11). The microvilli of this endothelium also show some fibrous‐looking attachments similar to those seen associated with the release of particles by the Type I vesicles (Figure 11D and 12A).

### 3.4 Conjunctiva Lining the Posterior Spectacular Space

The extension of the corneal epithelium into the peripheral conjunctiva consists of two cell layers with very numerous microvilli with similar dimensions to those over the outer surface of the cornea. Beneath the epithelium and basement membrane are somewhat randomly oriented bundles of large collagen fibrils with a diameter of 217.8 ± 104 nm (range between 63.4 and 422.5 nm) and which are significantly (*p* < 0.00001) larger than the collagen fibres of the cornea of *A. apraefrontalis*, in which the collagen fibre diameter is 27.5 ± 2.8 nm. These large fibres are part of the sclera and were used to confirm that the conjunctival observations were made beyond the corneal limbus.

**Figure 13:**
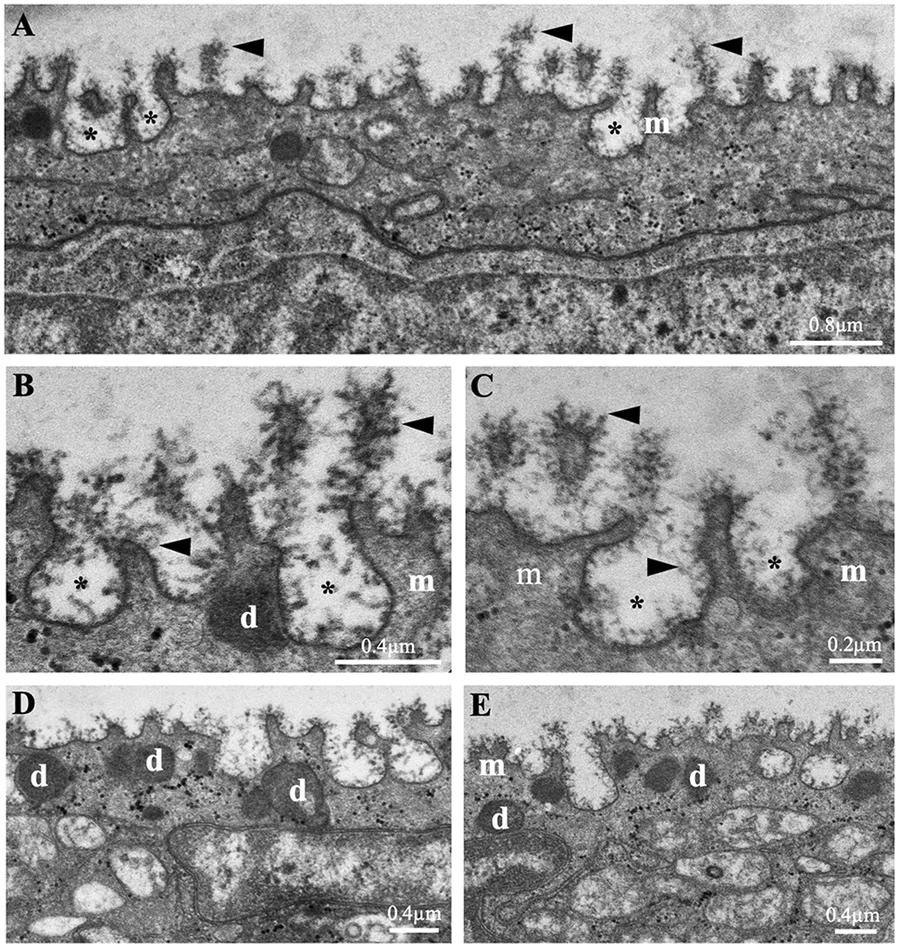
(A) Type I vesicles (asterisks) of the conjunctival epithelium lining the posterior spectacular space, which are open to the surface after expelling their contents (black arrowheads) that appear to line the open vesicles and cover the microvilli. (B and C) Close ups of open vesicles with dense aggregations of small particles (black arrowheads) adhering to the vesicle lining and the microvilli. Note the single dark Type II vesicle (d) in (B). (D and E). Mixture of Type I and darker Type II (D) vesicles. There are a number of Type I vesicles in the posterior region. m, microvilli.

As in the anterior conjunctiva, regularly‐spaced areas of reduced electron density, 80 to 95 nm in diameter, are present in association with breaks in the basal epithelial cell membrane in some regions. It appears that these structures are more common in the cells with numerous vesicles. In some other areas, there appear to be small breaks in the same region of the cell membrane without the lucent areas. Within some of the superficial epithelial cells are membrane‐bound vesicles, indicating that this posterior epithelium also has a secretory function. Similar to the conjunctiva lining the spectacle, two types of vesicles are characterised on ultrastructural features:

1. Type I vesicles with the mottled (non‐homogenous) contents seen in the spectacular conjunctiva are also quite numerous in the posterior conjunctiva (Figures 9D,E, and 13). These have a diameter of 311.3 ± 62.4 nm, which is similar (*p* = 0.149) to the equivalent spectacular vesicles. In addition, there are many electron‐lucent vesicles with a diameter of 326.6 ± 74.6 nm, which is not significantly different (*p* = 0.381) from the mottled vesicles and many of these are open and appear to be releasing their contents into the sub‐conjunctival space (Figures 9D,E and 13). As in the spectacle, small particles (approximately 5 nm in diameter and up to 140 nm in length) are attached to the inner part of the vesicles, to the outer surface of the epithelial cells and to the microvilli (Figures 9D,E and 13). These cells with open vesicles appear to be scattered throughout the epithelium and always appear to be surrounded by cells without vesicles.
2. The darker, more osmiophilic (Type II) vesicles seen in the spectacular conjunctiva component are only occasionally seen in the corneal conjunctiva (Figure 13B,D,E). They are smaller (296.7 ± 103.1 nm, *p* = 0.0033) than those in the anterior conjunctiva and are seldom seen discharging their contents. A few of the lighter (more electron‐lucent) vesicles, which are slightly larger (approximately 400 nm) than the darker vesicles, are present but are not seen discharging their contents.

**Figure 14:**
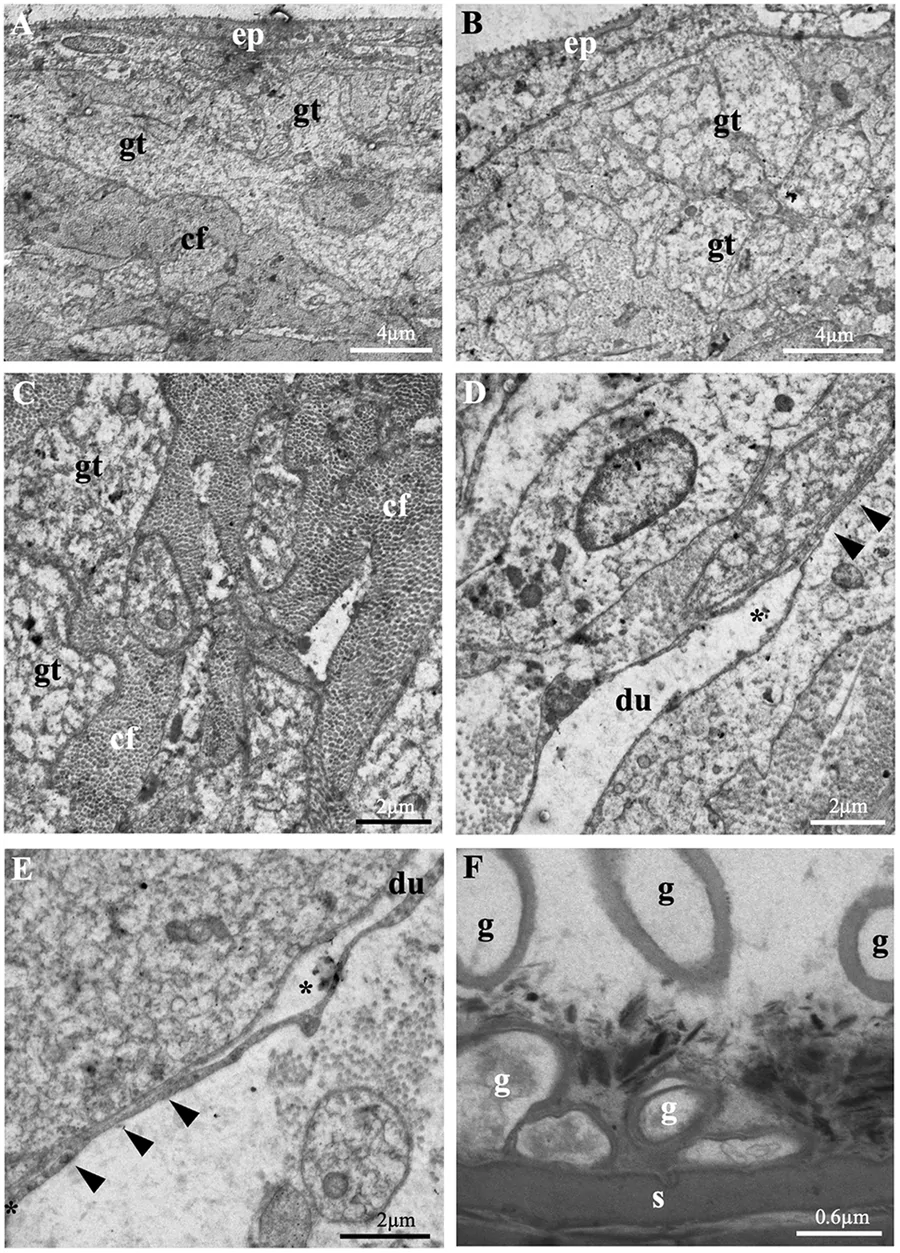
(A–C) Collections of pale cells filled with electron lucent vesicles, which resemble glandular tissue (gt) located beneath the epithelium (ep) of the conjunctiva lining the posterior spectacular space. Note the dense aggregations of collagen fibrils (cf). (D and E) Electron micrographs of large cell‐lined ducts (du) beneath the epithelium of the conjunctiva. Note the constriction of the ducts (arrows) and areas that widen (asterisks). (F) Aggregation of granules (g), similar to those typically found within the Harderian gland, adherent to the spectacle (s).

In the connective tissue beneath the epithelium of the conjunctiva lining the posterior spectacular space are several large collections of pale cells filled with vesicles, which are almost completely electron lucent. These vesicles (with a diameter of 799 ± 142 nm) are much larger than any of the vesicles described within the epithelium or endothelium. These cells resemble glandular tissue in the conjunctiva (Figure 14A–C).

Two large cell‐lined canals or ducts measuring up to 3 µm in width are present within this region of the conjunctiva (Figure 14D,E). In each duct, there are regions of constriction, about 3 µm in length, in which the central section (1.5 µm) is completely closed. On both ends of this closed region, the lumen opens to 45 nm for around 0.75 µm and then widens further (Figure 14D,E). These ducts may emanate from the Harderian gland, although typical Harderian granules were not present. However, various granules similar to those found in the Harderian gland of other species are present (Figure 14F) but they are scattered in the tissues and may have come from the sub‐spectacular space, the duct or be the result of damage to the Harderian gland during the enucleation of the eyes and subsequent surgery prior to preservation. These granules are largely electron lucent and may be in various stages of losing their contents (Figure 14F).

## 4 Discussion

### 4.1 Comparative Ultrastructure of the Snake Spectacle

The only ultrastructural studies of the snake spectacle to date are of the ball python *Python regius* (Da Silva et al. 2014) and 14 species of snakes from the families Boidae, Colubridae and Pythonidae (Da Silva et al. 2016), although only three transmission electron micrographs are included in these studies. However, the basic structure of the spectacle of the short‐nosed sea snake *Aipyusurus apraefrontalis* is similar to that described in these earlier studies, except for the dimensions of the different structural components.

The indentations in the keratinised surface of the spectacle have been described previously in the spectacle of four terrestrial and five aquatic snake species (Collin et al. 2025) and are similar to those found in the skin of at least 128 of 332 species examined by Arrigo et al. (2019) and in the skin of at least 23 of 27 fossorial species examined by Gower (2003). The diameter of these indentations or micropits in the surface of the spectacle of *A. apraefrontalis* ranged from 106 to 339 nm, while the mean diameter of indentations described in the spectacle of nine species of snakes (both sea and land) ranged from 42.4 to 182.6 nm (Collin et al. 2025) and was found to be 179 nm in diameter in *Morelia spilotes* (Campbell et al. 1999). The depth of the indentations in *A. apraefrontalis* (up to 120 nm) is much greater than that found in the spectacle of the Australian carpet snake *Morelia spilotes* (6.3 nm, Campbell et al. 1999). The prime function of the indentations in the skin is still a matter of conjecture. However, they are thought to hold dirt (Figure 3A) and other debris in order to increase friction and thus assist the snake's ability to move (Arnold 2002), although it has also been suggested that they confer hydrophobicity and therefore increase dirt‐shedding (Gower 2003; Arrigo et al. 2019).

The morphology of the epithelium of the spectacle in A. *apraefrontalis* is also similar to previous studies (Da Silva et al. 2014, 2016) but, due to the limited number of animals and the unknown stage of ecdysis, we did not observe the level of variability in epithelial thickness previously reported (Da Silva et al. 2016). However, the thickness of the basement membrane of *A. apraefrontalis* (107 ± 43 nm) is considerably greater than the thickness described in previous studies (mean of 40 nm, Da Silva et al. 2014, 2016).

The stromal collagen fibrils of *A. apraefrontalis* are consistent in diameter and separation throughout the thickness of the stroma, which accounts for the corneal transparency (Maurice 1957). However, the mean diameter of the stromal collagen fibrils of the spectacle (39.1 ± 5.9 nm) is significantly larger than those of the cornea (27.5 ± 2.8 nm), but significantly (*p* < 0.00001) smaller than the somewhat randomly arranged fibrils within the surrounding skin (87.5 ± 21 nm) or within the scleral eyecup (217.8 ± 106.6 nm), both of which are opaque. The basement membrane of the endothelium in *A. apraefrontalis* measured 44.5 ± 9.3 nm and does not resemble Desçemet's membrane. The posterior endothelium of the spectacle comprising a single cell layer in *A. apraefrontalis* (thickness of 1.29 ± 0.08 µm) has also been reported to be monocellular and poorly differentiated in the grass snake, *Natrix natrix* (Pchelyakov 1982). It has a thickness ranging between 1.6 and 3.3 µm across a large number of species examined by Da Silva et al. (2014; 2016).

Da Silva et al. (2014) found numerous vesicles lining the inner border of the posterior endothelial cells of the spectacle in the ball python, *Python regius*, and confirmed this in several other species of snakes (Da Silva et al. 2016). However, they were unclear as to the exact location of the vesicles, which we suggest were located in the peripheral transition region or in the conjunctival tissue, rather than behind the spectacle. We found no vesicles in the posterior endothelium of *A. apraefrontalis*, although vesicles are abundant in the conjunctival endothelium.

### 4.2 Comparative Ultrastructure of the Snake Cornea

In 1982, Pchelyakov carried out one of the few transmission electron microscopic studies of the cornea in snakes. This study focussed on the grass snake *Natrix natrix*, and reported an epithelium and an endothelium, both consisting of unicellular sheets of squamous cells. A substantia propria of the cornea was described but there was no report of either a Bowman's layer or Desçemet's membrane. Similar findings are reported here for *A. apraefrontalis*. Pchelyakov (1982) claimed that in the 3‐week embryo of *Natrix natrix*, the corneal epithelium has two layers; however, in the adult, the epithelium is described as a “unicellular sheet”. Walls (1942) and Duke‐Elder (1958) also claimed that the cornea in orphidians (group of squamate reptiles including modern snakes and reptiles more closely related to snakes than to other living groups of lizards) has a delicate single layer of epithelium. In *A. apraefrontalis*, the corneal epithelium has two layers, which would mediate a slightly higher level of biochemical exchange of oxygen, nutrients and metabolic products (Collin and Collin 2006).

The numerous microvilli on the anterior surface of the corneal epithelium in *A. apraefrontalis*, which are similar to those present in the majority of terrestrial and aerial vertebrates (Collin and Collin 2000, 2006), also have important functions. In species not within the Serpentes (the suborder to which snakes belong), which mostly lack a spectacle, the primary function of the corneal microvilli is thought to stabilize the tear film and minimise evaporation, thereby maintaining a smooth corneal surface and a clear retinal image. However, although the role of the dense aggregations of microvilli over the endothelium of the spectacle and the epithelium of the cornea (Figures 7 and 8), where evaporation is unlikely to occur, is unclear, there is still an obvious need to increase the surface area of the epithelial cell membranes to aid in intra‐ and extracellular movement of nutritional and waste products (Collin and Collin 2000).

The thickness of the snake cornea has been reported from light micrographs by several authors. The findings vary greatly and range from 48 to 451 µm (Da Silva et al. 2020), depending on the species and the age (size) of the snakes. The thickness of the cornea in *A. apraefrontalis* (42.1 µm in the centre and around 135.7 µm in the periphery) is within this range. Most authors agree that the epithelium of snakes has a basement membrane but there is no Bowman's layer (Pchelyakov 1982; Duke‐Elder 1958), which is also confirmed in *A. apraefrontalis*. The structure of the corneal stroma of *A. apraefrontalis* is very similar to the corneas of many other vertebrate species, with keratocytes squeezed between the lamellae and some lamellar branching and anastomosing. The thickness of the collagen fibrils (27.5 ± 2.8 nm) is similar to reports of 23.5 ± 18 nm in *Thamnophis sp*. (Craig and Parry 1981) and 30 nm in several snake species (Da Silva et al. 2016). Posterior to the stroma is a thin basement membrane, which separates it from a monolayered endothelium. Some authors have claimed that there is a Desçemet's membrane in the snake cornea (Oppal 1913; Millichamp 2022) but this is not substantiated in this study. In *A. apraefrontalis*, the thickness of the endothelial basement membrane is only 96.8 ± 19.5 nm, whereas in other vertebrate species, the thickness of Desçemet's membrane varies from 3−12 µm in humans (Gipson 1994), from 1−20 µm in mammals (Hayashi et al. 2002) and up to 1.47 µm in the little penguin (Collin and Collin 2000). Furthermore, a true Desçemet's membrane has a banded anterior portion (100 to 110 nm in humans, Jakus 1956; Gipson 1994) and the membrane increases in thickness with age (Murphy et al. 1984; Collin and Collin 2000). The basement membrane of the snake corneal endothelium does not meet any of these criteria and, hence, is not considered a Desçemet's membrane.

Microvilli are not found on the posterior surface of the corneal endothelium in *A. apraefrontalis*. Abdelftah et al. (2024) claimed that the corneal endothelial surface of *Python molurus* is covered with microvilli; however, these authors misinterpreted the work of Da Silva et al. (2014), who also did not find corneal endothelial microvilli but described microvilli over the inner (posterior) surface of the spectacle of *Python regius*.

### 4.3 Comparative Ultrastructure of the Snake Conjunctiva and Its Role in Secretion

It has long been claimed that in snakes, the cellular lining of the sub‐spectacular space is secretory (Kohl and Erster Thiel 1892; Muhse 1903; Bellairs and Boyd 1947; Da Silva et al. 2014, 2016). Millichamp (2022) also claimed that some of the epithelium of the sub‐spectacular space may be secretory, referencing both Bellairs and Boyd (1947) and Taub (1966). However, Taub makes no mention of epithelial secretory function in his study. There is also very little physical or pictorial evidence to support these claims. In Cuvier's earth snake *Rhinophis philippinensis*, Bellairs and Boyd (1947) found a well‐developed ‘sub‐brillar’ space, lined by epithelium of a secretory type, while in the sunbeam snake *Xenopeltis unicolor* and the red‐tailed pipe snake *Cylindrophis ruffus*, plaques of secretory epithelium were described. In 1982, Saint‐Girons published light microscopic evidence of glandular crypts in the angle of the conjunctival sac of the spotted blind snake, *Typhlops punctatus*(Saint‐Girons 1982). Pchelyakov (1982) also claims that in the embryo of the grass snake *Natrix natrix*, there are many vacuoles and microvesicles in the peripheral corneal epithelium. However, in the accompanying transmission electron micrographs, no vacuoles or microvesicles can be seen and there is no mention of their function.

In recent years, with more advanced techniques, several authors have claimed that vesicles are present in either the inner endothelium of the spectacle or the outer epithelium of the cornea, both of which line the sub‐spectacular space in snakes. However, there still remains a paucity of evidence and this appears to be confined to only two transmission electron micrographs in the studies by Da Silva et al. (2014, 2016) which show an occasional vesicle in the basal cells of the outer (anterior) epithelium of the spectacle and along the peripheral border of the inner (posterior) endothelium of the spectacle in the ball python, *Python regius* (Da Silva et al. 2014). They also reported small fluid‐filled vesicles along the “free border of the inner endothelium” of the spectacle, that is, facing the sub‐spectacular space, in a range of other species from three families (Boidae, Colubridae and Pythonidae), and moderately electron‐dense vesicles in the inner endothelium of the spectacle of the Siberian rat snake *Elaphe schrenckii* (Da Silva et al. 2016).

Da Silva et al. (2014) found that the structure of the inner endothelium of the spectacle of the ball python *Python regius* was consistent centro‐peripherally, including throughout the peripheral transition zone. However, Da Silva et al. (2016) claimed that these fluid‐filled vesicles are present in the central spectacular endothelium and assumed that the density of vesicles and microvilli diminishes throughout the transition zone. This is contrary to our findings in *A. apraefrontalis*, in that the central spectacular endothelium appears normal, that is, without vesicles, and the cells with secretory vesicles are only found in the peripheral conjunctival endothelium, at and beyond, the transitional zone. Again, contrary to the assumption made by Da Silva et al. (2016), the density of the microvilli on the conjunctival surface appeared to be similar to that of the central spectacular endothelium.

The vesicles found in the cellular layers lining the sub‐spectacular space in *A. apraefrontalis* have different electron densities and degrees of homogeneity. Secretory granules, which appear homogeneous and of low to medium electron density are typical of mucous granules (Minucci et al. 1992) and may be serous, seromucous or mucous secretions (Chieffi et al. 1992). Some of the vesicular heterogeneity may indicate the different stages of condensation of the polymers within the vesicles (Verdugo 1990). Based on the ultrastructure of the vesicles and the expelled material, there are vesicles with differing contents and methods of release, nominally defined here as Types I and II.

The mottled (non‐homogenous) Type I vesicles in *A. apraefrontalis* contain short fibres (approximately 5 nm diameter and up to 140 nm in length), which resemble mucin fibrils. The thickness and length of individual polymers of mucin present in mucus can vary greatly in different regions of the body and in different species. According to Berry et al. (2000), the unique characteristics of normal human secreted ocular mucins are their wide size range and short oligosaccharide side chains. In humans, the diameter of the molecules of ocular mucin approximates 1 nm (Berry et al. 2001; Round et al. 2002), although the diameter of human cervical mucins may range from 5 nm (Sheehan et al. 1986) to over 500 nm due to aggregation of the fibres (Brunelli et al. 2007). Porcine gastric mucin is 5 nm in diameter (Sheehan et al. 1986). The length of mucin macromolecules can vary from 36 nm (Round et al. 2002) to in excess of 3 µm (McMaster et al. 1999; Round et al. 2002) or even 5 µm (Bansil et al. 1995). It is clear that our findings for the dimensions of the fibrillar structures being expelled from the Type I vesicles in both the posterior endothelium of the spectacular conjunctiva and the anterior epithelium of the corneal conjunctival epithelium fall well within the range reported for mucin molecules.

The contents of the epithelial and endothelial vesicles appear to be large polymers, which are concentrated or condensed (Verdugo 1990). The mechanism of release of the contents from vesicles involves the serous secretory granules docking with the plasma membrane and opening pores, allowing rapid release of the contents, which become hydrated and show extensive expansion to form transportable mucus. This mechanism has been called ‘the Jack‐in the‐box mechanism of exocytosis' (Verdugo 1990) and is clearly demonstrated in the vesicles in the conjunctival endothelium of the spectacle of *A. apraefrontalis* (Figures 11 and 12).

In the large paler (less electron dense) Type II vesicles, the release process appears to involve this more explosive discharge and the filamentous structures may be actin filaments (Figure 12). The diameter of an actin filament is 5 nm (Lentz 1971), which is similar to the diameter of the filaments seen in *A. apraefrontalis* (5.7 ± 2.3 nm). Filamentous actin is known to be involved in a variety of intracellular activities, including propelling bacteria to the cell periphery prior to membrane protrusion (Dhanda et al. 2020) and assisting in the motility of endocytic vesicles (Merrifield et al. 1999) and other membrane organelles (Giardini et al. 2003). Actin is also associated with secretory cells (Nakata and Hirokawa 1992; Holz and Axelrod 2022) and facilitates the release of the contents of vesicles (Berberian et al. 2009). In *A. apraefrontalis*, the lack of any actin filaments within the material discharged into the sub‐spectacular space (Figures 11 and 12) indicates that these filaments may be active components that reside in these cells. The compression forces balancing actin gel expansion are predicted to squeeze the vesicles (Giardini et al. 2003), which release their contents. The expulsion can be very rapid. In an in vitro study in the slug *Ariolimax columbianus*, Verdugo (1990) claims that the giant secretory granules show a 400‐ to 600‐fold volumetric expansion over just 20 msec. However, in an earlier study, the granules were found to expand to about 25 times their volume in 66 msec before bursting (Verdugo et al. 1987).

### 4.4 The Role of Ducts

Walls (1942) claimed that there is a single duct from the Harderian gland to the sub‐spectacular space in the eyes of snakes. Bellairs and Boyd (1947) found that in “typical” snakes (e.g., colubrids and vipers), the Harderian gland discharges via a single duct into the upper end of the lacrimal duct and has no direct communication with the sub‐brillar space, although in the sunbeam snake *Xenopeltis unicolor* and the red‐tailed pipe snake *Cylindrophis ruffus*, the Harderian gland discharges via two or more ductules. In the constrictors (e.g., *Constrictor [Boa] constrictor*, *C. rufus*), the Harderian gland discharges directly into the sub‐brillar space as well as into the lacrimal duct. This is echoed by Payne (1994), who also reported that the Harderian gland may open into the sub‐brillar space by several (separate) small ducts, with the main duct alone entering the lacrimal duct directly. Chieffi et al. (1992) found that in the green whip snake *Coluber viridiflavus*, the ducts of the Harderian gland are not numerous and are often close together but they did not comment on the destination of these ducts.

The large endothelial‐lined ducts of *A. apraefrontalis* in the conjuctiva may emanate from the Harderian gland indicating that there may be several ducts in this species. The absence of typical Harderian granules within these ducts could be due to loss during histological preparation or that the appearance and function of the granules may be species‐specific given the Harderian glands have been found to deliver fluid to non‐visual sense organs such as the vomeronasal organ (Rehorek 1997). Alternatively, the movement of granules through the duct may be controlled by the gland or the duct, which releases fluid and granules in phases according to the needs of the sub‐spectacular space and our tissue may have been obtained during an empty phase. The presence of regions of the ducts, which appear completely closed supports this concept of controlled delivery of fluid, mucous and possible Harderian granules (Figure 14D,E). Harderian granules were found associated with the tissues (Figure 14A–C) and may have come loose from damage to the Harderian gland during surgical enucleation of the eyes or the duct or from the sub‐spectacular space. The different electron densities of these granules may indicate that they are at different stages of release.

### 4.5 Non‐Harderian Glandular Tissue

In 1903, Muhse found that in the blind snake *Typhlops vermicularis*, thickening of the conjunctiva at the edge of the sac is due to the presence of gland cells (Muhse 1903). However, it is not entirely clear whether these cells are in the epithelium or in the connective tissue of the conjunctiva. In contrast to that observed in other snakes, Bellairs and Boyd (1947) reported that in the red‐tailed subspecies of constrictors *Boa constrictor constrictor* and the northern eyelash boa *Trachyboa boulengeri*, there are detached islets of secretory tissue and single acini (distinct from the Harderian gland), which discharge directly into the sub‐brillar space. They also describe a ring of secretory tissue in the keeled slug snake *Pareas (Amblycephalus) carinatus*, which is continuous with the anterior Harderian gland acini, completely surrounds the upper end of the lacrimal duct and the anterior part of the sub‐brillar space and discharges extensively into both of them. The glandular tissue described in *A. apraefrontalis* does not resemble the Harderian gland in structure and hence, may be a detached eyelet or an isolated secretory gland.

### 4.6 Are Lipids Involved?

Various lipids are found on the surface of the skin in snakes (Schell et al. 1985; Andonov et al. 2020) but there appears to be no evidence that any lipids are produced by glandular tissues associated with the eye. Walls (1942) suggests that an oily secretion from the Harderian gland is produced to lubricate the sub‐spectacular space to assist eye movements beneath the stationary spectacle. Duke‐Elder (1958), who cites Bellairs and Boyd (1947, 1950), claims that an oily secretion from the Harderian gland flows into the closed conjunctival sac (sub‐spectacular space) but neither of the two studies by Bellairs and Boyd mentions the secretion as being oily.

More recently, Schwab (2012) states that, in snakes, the lacrimal glands are absent and that an oily secretion from their Harderian glands flows into the closed conjunctival sac; however, he gives no indication of the source of this information. Several authors claim that among snakes, tears, which are predominantly serous, are produced by the accessory lacrimal (Harderian) gland and flow into the sub‐spectacular space (the sub‐brillar space) (Taub 1966; Saint‐Girons 1982; Souza et al. 2015; Hellebuyck and Solanes Vilanova 2023). In addition, the Harderian gland in both water and garter snakes is claimed to be muco‐serous and that lipid is not among the secretory products of this gland in reptiles (Taub 1966; Paule 1957; Payne 1994). Similarly, Cheiffi et al. (1992) claim that there is no lipid in the Harderian gland of snakes and that the secretory product can be serous, seromucous or mucoserous.

The presence of what appear to be fibres being expelled from the vesicles in both the posterior conjunctival epithelium and the anterior conjunctival endothelium of the sub‐spectacular space is suggestive of the presence of protein chains rather than lipid. The thickness of the fibres is approximately 5 nm in the posterior conjunctival epithelium and 5.7 nm in the anterior endothelium, which is similar to the thickness of individual polymers of mucin found in other species or sites. In humans, the diameter of ocular mucin is approximately 1 nm (Berry et al. 2001; Round et al. 2002), although the diameter of human cervical mucins may range from 10–500 nm due to fibre aggregation (Brunelli et al. 2007).

### 4.7 The Function of Secretory Tissue

The function of fluid in the sub‐spectacular space, whether from the Harderian gland or from serous or mucous secretions from the cellular lining is not well understood. Walls (1942) suggests that lubrication is needed within the sub‐spectacular space to assist smooth eye movements and that snakes ‘elected the Harderian gland simply because of the superiority of oil over water as a lubricant’. However, there is no evidence of the presence of any lipids in the sub‐spectacular space. Similarly, Chieffi et al. (1992) claims that the most primitive and most likely function of the Harderian gland is lubrication of the eye. However, few of those reporting the presence of secretory epithelia appear to have considered the function of these secretory cells. In snakes, the greater part of the Harderian secretion is diverted from the sub‐brillar space into the lacrimal duct and in many burrowing forms, where the circulation in the tiny sub‐brillar space is minimal, all the secretion of the enormous Harderian gland passes directly into the lacrimal duct (Bellairs and Boyd 1950). In such a case, the secretions of the cells lining the conjunctival sac could either augment the secretions from the Harderian gland or fill the sub‐spectacular space.

Rehorek et al. (2003) reported that, despite recent assertions that in snakes the Harderian gland may function as part of the vomeronasal system, the function of the Harderian gland and its mucous secretions remains unknown. Da Silva et al. (2016) suggested that the microvilli and vesicles in the inner endothelium of the spectacle are responsible for maintaining the relative hydration of the spectacle and thereby its transparency. The mechanism by which the endothelium of the cornea removes fluid from the stroma is referred to as the endothelial pump, whereby ions are transported across the endothelium and this generates an osmotic gradient to draw water from the stroma (Edelhauser et al. 1994). It is assumed by Da Silva et al. (2016) that a similar process occurs in the snake spectacle to control hydration and maintain transparency. However, the vesicles found in the spectacular endothelium of *A. apraefrontalis* are conjunctival, and hence peripheral to the spectacle and would not be expected to have an effect on transparency.

Da Silva et al. (2016) consider that pinocytosis is part of the mechanism maintaining transparency of the spectacle, but the diameter of the vesicles they report in the spectacular endothelium (220 to 430 nm) appears to be much greater than the diameter of pinocytotic vesicles, which transport various molecules across an endothelium and break through the cell membrane. Pinocytotic vesicles are typically 74 ± 3.5 nm in diameter in human corneal endothelium (Rates et al. 2022), 75.7 ± 1.53 nm in diameter in various other endothelia (Casley‐Smith 1969) or are between 60 and 80 nm, depending upon the endothelial type and fixation procedures (Mazzone and Kornblau 1981). The diameters of the vesicles in the peripheral endothelium of the spectacle in *A. apraefrontalis* (range from 159 to 450 nm for Type I and from 170 to 588 nm for Type II vesicles) are similar to those described by Da Silva et al. (2016); however, they are much larger than pinocytic vesicles, which would appear to negate their role in pinocytosis to contribute to spectacle transparency. Da Silva et al. (2016) also claim vesicles were present in the central region of the endothelium of the spectacle and assumed that the density of the vesicles diminishes throughout the transition zone to the conjunctiva, ‘much like the stroma loses it organizational structure, as there is no longer a need to uphold transparency’. This is precisely the opposite to our finding that the central endothelium of the spectacle appears devoid of vesicles and their presence is confined to the transitional zone and conjunctiva, where the need for transparency is not as critical.

## 5 Conclusions

Little is known of the role of the sub‐spectacular space or its contents in snakes other than potentially lubricating the opposing surfaces of the overlying and immobile spectacle and the underlying cornea, which is continuous with the scleral eyecup containing the neural retina and responsible for visual transduction. The ultrastructure of the spectacle and cornea is described for the first time in *Apysurus apraefrontalis* revealing that the peripheral (conjunctival) lining of the sub‐spectacular space is populated by high densities of microvilli interspersed with at least two types of secretory vesicles, which appear to deliver serous and probably mucous secretions via different secretory mechanisms. Ducts and glandular tissue found within the conjunctiva also indicate that there are other mechanisms involved (including the Harderian gland) in delivering substances into this crescent‐shaped sub‐spectacular or sub‐brillar space and maintaining its integrity to ensure the eye is able to optimise its refractive power and still move freely.

## Author Contributions

**H. Barry Collin:** conceptualization, investigation, writing – original draft, methodology, formal analysis, data curation, validation, visualization, writing – review and editing. **Boyin Liu:** methodology, visualization. **Sarah Wilson:** methodology, visualization. **Jenna M. Crowe‐Riddell:** investigation, methodology, visualization, resources, writing – review and editing, project administration, funding acquisition. **Shaun P. Collin:** conceptualization, investigation, funding acquisition, writing – review and editing, methodology, validation, visualization, resources, project administration.

## Conflicts of Interest

The authors declare no conflicts of interest.

## Data Availability

The data that support the findings of this study are available on request from the corresponding author. The data are not publicly available due to privacy or ethical restrictions.
